# Development and initial validation of a multivariable predictive Early Adversity Scale for Schizophrenia (EAS-Sz) using register data to quantify environmental risk for adult schizophrenia diagnosis after childhood exposure to adversity

**DOI:** 10.1017/S0033291722001945

**Published:** 2023-08

**Authors:** Patsy Di Prinzio, Jonas Björk, Giulietta Valuri, Taryn Ambrosi, Maxine Croft, Vera A. Morgan

**Affiliations:** 1 Neuropsychiatric Epidemiology Research Unit, School of Population and Global Health, The University of Western Australia, Perth, Australia; 2 Division of Occupational and Environmental Medicine, Lund University, Lund, Sweden; 3 School of Population and Global Health, The University of Western Australia, Perth, Australia

**Keywords:** Childhood adversity, epidemiology, prediction, psychosis, ranking, risk, scale, schizophrenia

## Abstract

**Background:**

Additional to a child's genetic inheritance, environmental exposures are associated with schizophrenia. Many are broadly described as childhood adversity; modelling the combined impact of these is complex. We aimed to develop and validate a scale on childhood adversity, independent of genetic and other environmental liabilities, for use in schizophrenia risk analysis models, using data from cross-linked electronic health and social services registers.

**Method:**

A cohort of *N* = 428 970 Western Australian children born 1980–2001 was partitioned into three samples: scale development sample (*N* = 171 588), and two scale validation samples (each *N* = 128 691). Measures of adversity were defined before a child's 10th birthday from five domains: discontinuity in parenting, family functioning, family structure, area-level socioeconomic/demographic environment and family-level sociodemographic status. Using Cox proportional hazards modelling of follow-up time from 10th birthday to schizophrenia diagnosis or censorship, weighted combinations of measures were firstly developed into scales for each domain, then combined into a final global scale. Discrimination and calibration performance were validated using Harrell's C and graphical assessment respectively.

**Results:**

A weighted combination of 42 measures of childhood adversity was derived from the development sample. Independent application to identical measures in validation samples produced Harrell's Concordance statistics of 0.656 and 0.624. Average predicted time to diagnosis curves corresponded with 95% CI limits of observed Kaplan–Meier curves in five prognostic categories.

**Conclusions:**

Our Early Adversity Scale for Schizophrenia (EAS-Sz), the first using routinely collected register data, predicts schizophrenia diagnosis above chance, and has potential to help untangle contributions of genetic and environmental liability to schizophrenia risk.

## Introduction

A child's genetic inheritance contributes to their risk of developing schizophrenia, a highly polygenic disorder, with heritability estimated at 60–80% (Owen, Sawa, & Mortensen, 2016). Lifetime morbid risk ranges from approximately 1% within the general population to a concordance of 48% for monozygotic twins where one twin is affected (Gottesman, 1994; Gottesman & Erlenmeyer-Kimling, 2001; Gottesman, McGuffin, & Farmer, 1987). Environmental exposures are also implicated in schizophrenia risk, either exclusively or in interaction with genetic risk (Morgan & Gayer-Anderson, 2016; van Os, Kenis, & Rutten, 2010; Varese et al., 2012). Environmental exposures previously studied include physical trauma such as obstetric complications (Cannon, Jones, & Murray, 2002), cannabis use (Marconi, Di Forti, Lewis, Murray, & Vassos, 2016) and exposure to a wide range of experiences termed ‘childhood adversity’ (Morgan & Gayer-Anderson, 2016; van Os et al., 2010; Varese et al., 2012). The latter include discontinuity in parenting either by death or separation (Morgan et al., 2007; Paksarian, Eaton, Mortensen, Merikangas, & Pedersen, 2015a); childhood abuse (Read, van Os, Morrison, & Ross, 2005; Varese et al., 2012); social disadvantage (Wicks, Hjern, & Dalman, 2010); being born or raised in an highly urbanised environment (Vassos, Pedersen, Murray, Collier, & Lewis, 2012); migration; and minority ethnicity (Cantor-Graae & Pedersen, 2013; Morgan, Charalambides, Hutchinson, & Murray, 2010), including Indigenous status (Tapsell, Hallett, & Mellsop, 2018). Childhood exposure to maladaptive family functioning has been associated with a broad range of psychopathology in adulthood (Kessler et al., 2010). While many factors appear to operate at the individual level, neighbourhood (or area) level characteristics such as disadvantage, residential mobility and crime rates have also been found to impact on risk (Veling, Susser, Selten, & Hoek, 2015).

Modelling adversities and their interaction with genetic liability in risk factor analysis is complex. Severity of impact will vary depending on the adversity and may be related to age at exposure (Agid et al., 1999; Paksarian et al., 2015a; Paksarian, Eaton, Mortensen, & Pedersen, 2015b). Risk may increase in a dose–response relationship as the number of adversities increases (Morgan et al., 2008a; Wicks, Hjern, Gunnell, Lewis, & Dalman, 2005; Zammit, Lewis, Dalman, & Allebeck, 2010) and, because adversities cluster (Dong et al., 2004; Kessler et al., 2010), a comprehensive measure of early life adversity exposure should account for the interactive effects of co-occurring adversities on the total risk for outcome (Evans, Li, & Whipple, 2013). To date, we have only simple tools for measuring relative contributions in risk attributable to given sets of childhood adversities, and for advancing our understanding of how they may interact with genetic liability.

A common approach to modelling adversities in the study of schizophrenia has been to include them as separate terms in multivariable explanatory models (Morgan et al., 2019; Veling et al., 2015) with some studies allowing for the possibility of interactions between risk factors (Zammit et al., 2010). Some summary exposure scores have also been proposed. One approach is to sum the number of risk exposures (Veling et al., 2015), which implicitly assumes that the risk contribution of each exposure is equal. Other approaches apply weightings reflecting the severity of impact (determined from estimates in literature) to exposures before summing (Padmanabhan, Shah, Tandon, & Keshavan, 2017; Vassos et al., 2020). More complex summaries that take interactions into account have been developed to predict several more common adult mental disorders, although not schizophrenia (Green et al., 2010; Kessler et al., 2010). One prognostic scale recently developed is the Exposome Score for Schizophrenia (Pries et al., 2019). Here, candidate models, incorporating severity weightings of eight exposures (cannabis use, winter birth, hearing impairment, childhood neglect or psychological, physical or sexual abuse, and childhood peer victimisation), were alternately derived via techniques including logistic regression. Testing and assessment conducted in an independent data set (Pries et al., 2020) indicated that models derived via logistic regression predicted schizophrenia at least as well as, or more accurately, than other techniques trialled, including those using machine learning.

Absent in the literature is a comprehensive adversity scale for risk of schizophrenia that considers correlations between variables, clustering of observations, possible interactions between effects, differences in severity of impact and differential impact depending on age at exposure. Critically, there is a need for a scale that can capture adverse exposures in childhood when, according to the neurodevelopmental/developmental perspectives on schizophrenia, it is assumed that the impact of stress on a rapidly developing brain may be greatest (Murray, Bhavsar, Tripoli, & Howes, 2017). Our aim was to fill this gap by developing and validating a childhood adversity scale covering multiple domains of adversity with the goal of delivering such as a robust tool which could be further applied to risk analysis models. Moreover, because childhood exposures may occur up to two decades or more before schizophrenia onset, a tool was needed that could be applied to existing prospective data registers. The use of prospectively collected data has great advantages over retrospective, possibly biased recall data (Vargas & Mittal, 2018). Hence, our focus was on developing a scale for use with cross-linked data from multiple electronic health and social services registers. Finally, to maximise translational capacity, the methodology needed to be transparent, easy to replicate and adaptable to variations in specific register data.

## Methods

### Cohort identification

We used a whole-population cohort of children (*N* = 428 970) [henceforth referred to as the e-Cohort (Morgan et al., 2011)] which included all children born alive in Western Australia between 1 January 1980 and 31 December 2001, born to two sets of mothers: (i) those with no recorded contact with mental health services (*N* = 407 639 children, *N* = 217 929 mothers); and (ii) those with a diagnosis of psychotic illness (*N* = 21 331 children, *N* = 10 605 mothers) before 31 December 2015. No data were provided to the research team on mothers on the registers with a non-psychotic psychiatric illness diagnosed before 31 December 2015, nor on their children [estimated to be approximately 61 000 mothers and 115 000 children (Justice, 2022)], as they were excluded from the original ethics agreement covering the project. Children of the e-Cohort were identified on the Midwives' Notification System (Gee & Dawes, 1994), which includes mandatory, prospectively collected data on all infants born in Western Australia at 20 weeks gestation or greater or weighing at least 400 g, including home births, and not restricted to live births. Mothers with a psychotic illness were sourced through linkage of records on the Midwives' Notification System to the Hospital Morbidity Data Collection and Mental Health Information System which covers records of all public and private inpatient hospital admissions, as well as public outpatient and ambulatory care contacts with mental health services across the State, dating back to 1966. Psychotic illness was broadly defined using ICD-9 codes 295–298 to include schizophrenia (ICD-9 295; *N* = 1220 mothers), affective psychoses (ICD-9 296; *N* = 8365 mothers), paranoid states and other non-organic psychoses (ICD-9 297 and 298; *N* = 1020 mothers). ICD-8 and ICD-10 equivalents, used in a minority of records, were mapped to ICD-9. For those fathers named on the Midwives' Notification System, relevant covariate data were also extracted from linked State registers. The full cohort of children, their mothers and fathers named on the Midwives' Notification System were linked to electronic mental and physical health data, mortality, child protection and corrective services records. Linkage was carried out by the Data Linkage Branch of the Western Australian Department of Health. Full details on the linked registers used to establish and characterise the e-Cohort have been published previously (Di Prinzio et al., 2018; Morgan et al., 2011).

This study was approved by the Western Australian Department of Health Human Research Ethics Committee (2011/75) and The University of Western Australia Human Research Ethics Committee (RA/4/1/1322).

### Cohort partitioning

We randomly generated three non-overlapping partitions of children from the e-Cohort: (i) a 40% (*N* = 171 588) random sample to develop the computer algorithm, (ii) a 30% (*N* = 128 691) random sample to validate it and explore calibration, and (iii) a final 30% (*N* = 128 691) partition to repeat validation and calibration on a fresh sample.

### Outcome

*Children's follow up and schizophrenia diagnosis:* Children were followed up from their 10th birthday to 30 June 2015, their date of death or date of onset of schizophrenia, whichever came first. Children were defined as having schizophrenia if they had any recorded diagnosis of schizophrenia (ICD-9 295-all or ICD-10 F20, F21, F23.1, F23.2 or F25) in their mental health inpatient admissions or ambulatory/outpatient contacts; the date of the first record of schizophrenia was used as a proxy for date of onset. Information on child mortality was obtained from the Western Australian Death Registry.

### Measures of adversity exposure

Guided by literature, we considered the range of childhood adversities associated with schizophrenia. We calculated measures of such adversities where the scope and accuracy of register data provided sufficient information to do so. Complete descriptions are in the Covariate Dictionary in the online Supplementary materials. These measures were grouped into five broad domains of adversity, A1 to A5:

*A1 – Discontinuity in parenting:* Before the age of 10 years covered any separation of a child from a parent including *hospitalisation*, *parental death*, *placement in foster care* and *parental incarceration*. Separations were further categorised by the child's developmental epoch: (i) under 1 year of age, (ii) ages 1–4 and (iii) ages 5–9.

*A2 – Family functioning: Parental corrective services contacts* included detention, diversionary programmes and non-custodial orders. *Child protection contacts* were available from 1989 onwards and covered any notification of allegations of child abuse (sexual, physical and emotional) and neglect made to child protection services before a child's 10th birthday concerning the index child or any of their maternal siblings; notifications were further classified as *substantiated* or *unsubstantiated*.

*A3 – Family structure: Mother's age* and *paternal age* at the time of child's birth were calculated from birth records, as was *maternal marital status* and *child's birth order*. Calculation of the *size of the family* a child belonged to at the time of their 10th birthday was possible using Midwives Notification data up to 2001 and hospital morbidity records for maternal obstetric events after 2001.

*A4 – Area-level socioeconomic/demographic environment:* Area-level *socioeconomic disadvantage* and *geographical remoteness/level of urbanicity* of the mother's neighbourhood were determined from census data using the mother's address at the time of a child's birth. Additional area-level measures from census information reflected the proportion of persons within an area who were *Indigenous* (of Aboriginal or Torres Strait Islander descent), *Australian born*, *never married*, *living in one parent families*, *unemployed*, *living in a different residence to 5 years* previously, *living in a different residence to 1 year* previously, *living in semi-detached* dwellings, *living in flats*, *living in rented* dwellings, who had *no post school qualifications* or who *spoke a language other than English at home and did not speak English well*. An area-level measure of *ethnic heterogeneity* was calculated (Morgan et al., 2008b). Area-level *crime rates* based on the arrest rate per 1000 residents in 2002 were determined. The distribution of socioeconomic disadvantage within a postcode provided a measure of the geographical *variability of disadvantage* within a localised area.

*A5 – Family-level sociodemographic status:* A child's *father* was classified as *known* if he was named on the birth record. The *child's Indigenous* status was scored positive if the child and/or either parent was identified as Indigenous (of Aboriginal or Torres Strait Islander descent) in any of the data sources available. *Parental Australian state/territory of origin* and *migrant status* were recorded; the latter was categorised according to the affluence of the country of origin using World Bank rankings. *Father's occupation* was classified according to skill level, as defined by the Australian Bureau of Statistics.

Online Supplementary Table S1 details missing data. Child protection records were incomplete for children born prior to July 1989, affecting 70 474 children across the whole e-Cohort, and resulting in underestimation of derived exposures. Father's occupation status was missing for 3.7% of known fathers (6186/165 754). They were grouped with unemployed fathers for analysis. The level of missing data for remaining exposures was low (<1%) and considered unlikely to introduce bias.

### Statistical analysis

We used (i) the largest of our three non-overlapping partitions (40%) to develop the algorithm, (ii) one of the 30% partitions to validate and explore calibration, and (iii) the final 30% partition to repeat validation and calibration, and thus assess generalisation error (Hastie & Fieldman, 2009).

*Development:* We used a hierarchical approach to model associations between adversity exposures and schizophrenia diagnosis, using the 40% development sample of the e-Cohort. For each domain of adversity, we developed multivariable Cox models considering exposures specific to that domain only. This process was repeated independently for each domain in turn. The resultant models generated five adversity scores for each child, one for each domain (Equation 1). Next, these domain-level scores were used as input to a global multivariable Cox model which estimated their optimal combination to best explain the risk of schizophrenia diagnosis. Hence, a single score – the Early Adversity Scale for Schizophrenia (EAS-Sz) score – could be determined for each child (Equation 2).

To determine domain-specific models, we first considered the parsimonious parameterisation of potential covariates into discrete measures. This was guided by the assessment of estimated bivariate hazard ratios, and graphical assessment of the hazard rate shapes as illustrated by Nelson Aalen plots. Measures that displayed bivariate association with schizophrenia diagnosis of *p* ⩽ 0.2 were considered as candidates in further model development. Within a domain, all candidate exposures were evaluated in the presence of each other using augmented backwards elimination (Dunkler, Plischke, Leffondre, & Heinze, 2014) and a significance level of 5%. Clearly influential subjects based on visual assessment of likelihood displacement [v-martingale residual graphs (Hosmer, Lemeshow, & May, 2011)] were excluded. From the optimal domain model, each child's domain score was calculated as the sum of the estimated log hazard ratios for the exposures experienced (Equation 1).1
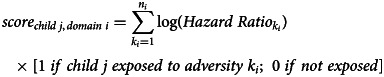
where

*i* = adversity domain = 1–5,

*j* = child = 1 to *n*_development sample_,

*n_i_* = number of terms in the optimal multivariable model for domain *i*.

The global model considered the five domain scores as continuous variables, each associated with risk of schizophrenia diagnosis. The model also considered the ability of any of the 10 two-way multiplicative interactions to improve the explanation of variation. Augmented backwards elimination and a significance level of 5% were used to determine which minimal set of interactions and raw domain scores were of optimal predictive benefit. Screening and exclusion of influential observations mirrored the domain-level approach. The EAS-Sz score is defined as the global score – a weighted combination of domain scores as per the preferred global model and is detailed in Equation 2.2
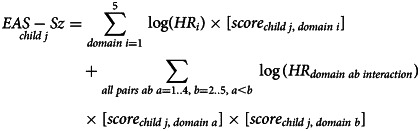
where

*i* = adversity domain = 1–5,

*j* = child = 1 to *n*_development sample_,

*score*_*child j*, *domain i*_ is as defined in Equation 1,

HR is estimated hazard ratio.

Throughout domain-level and global modelling processes, proportionality assumptions were assessed by χ^2^ tests using Schoenfeld residuals. The robustness of parameter estimates was investigated by comparing them with corresponding estimates determined from equivalent logistic regression models with log(time) offsets.

*Validations:* We used two separate 30% random samples of our cohort (each *N* = 128 691), which did not overlap with our development dataset or each other, to conduct validation assessments on domain and global models. To assess *discrimination* ability of our Cox model, we calculated Harrell's Concordance (Harrell, Califf, Pryor, Lee, & Rosati, 1982). For *calibration* assessment, we prepared graphical comparisons of predicted *v.* observed diagnosis prevalence over follow-up, across five risk stratification categories, using the methods of Royston (Royston, 2015). The categories were defined based on a pragmatic partitioning of the global adversity scores where their definition balanced the need for sufficient categories to provide useful discrimination, while maintaining stability of parameter estimates derived from such categories. Observed natural cut-points of the score's distribution were exploited where possible.

## Results

Our development dataset comprised *N* = 171 588 births. Children who died before age 10 years (*N* = 1258) were treated as not at risk of developing schizophrenia and were excluded. Of the remaining 170 330 children, 656 had at least one schizophrenia diagnosis before the end of follow-up. A further 967 children died before the end of follow-up and before receiving a diagnosis. Adversity exposure profiles for children excluded or censored due to death are detailed in online Supplementary Table S2. Loss to follow-up due to outmigration is assumed to be low (Morgan et al., 2011).

After assessment and relevant consolidation of 70 candidate measures, including some re-parametrisation of original versions to comprise fewer category classifications, 42 were found to have potential to be predictive of schizophrenia diagnosis: 15 in the discontinuity in parenting domain, two in family functioning, five in family structure, 16 in area-level socioeconomic/demographic environment and four in family-level sociodemographic status. The covariate dictionary in online Supplementary materials provides more details.

The proportion of children exposed to adversities ranged from 0.4% (having a mother incarcerated before the child's 10th birthday) to 97.3% (being born in an area with all but the lowest of crime rates – total annual crime arrests ⩾50 per 1000 persons) (Table 1). Corresponding proportions for validation datasets can be viewed in online Supplementary Table S3. Associations between adversity exposures and schizophrenia diagnosis are summarised in Table 1 and range from modest (either or both parents deceased before child's 10th birthday, HR = 1.39, 95% CI 0.77–2.53) to large (any placement in foster care before a child's 10th birthday, HR = 9.56, 95% CI 5.80–15.7).

**Table 1.** Exposure to adversity in development sample (number and percentage) and bivariate association with schizophrenia diagnosis (hazard ratios and 95% confidence intervals)

Adversity exposure	Child with diagnosis of Sz		Total development sample		Bivariate association	
	*N* = 656	%	*N* = 171 588	%	HR	95% CI
Discontinuity in parenting						
Mother hospitalised ⩾8 days when child aged 15 days to <1 year	16	*2.4*	1634	*1.0*	2.19	(1.33–3.60)
Mother hospitalised ⩾8 days when child aged 1 to <5 years	222	*33.8*	30 954	*18.0*	1.79	(1.52–2.10)
Mother hospitalised ⩾8 days when child aged 5 to <10 years	124	*18.9*	16 445	*9.6*	1.81	(1.49–2.20)
Child hospitalised ⩾8 days when child aged 15 days to <1 year	42	*6.4*	4131	*2.4*	2.72	(1.99–3.72)
Child hospitalised ⩾8 days when child aged 1 to <5years	44	*6.7*	4180	*2.4*	2.40	(1.77–3.26)
Child hospitalised ⩾8 days when child aged 5 to <10 years	19	*2.9*	1967	*1.1*	2.12	(1.34–3.34)
Father hospitalised ⩾8 days when child aged 0 days to <1 year	11	*1.7*	947	*0.6*	2.61	(1.44–4.74)
Father hospitalised ⩾8 days when child aged 1 to <5 years	38	*5.8*	4826	*2.8*	1.81	(1.31–2.51)
Father hospitalised ⩾8 days when child aged 5 to <10 years	42	*6.4*	6008	*3.5*	1.75	(1.28–2.39)
Deceased parents, either or both, before child aged <10 years	11	*1.7*	2065	*1.2*	1.39	(0.77–2.53)
Any placement in foster care before child aged <10 years	16	*2.4*	697	*0.4*	9.56	(5.80–15.7)
Any incarcerations for mother when child aged <10 years	11	*1.7*	649	*0.4*	5.30	(2.92–9.62)
Any incarcerations for father when child aged 0 days to <1 year	12	*1.8*	884	*0.5*	4.19	(2.36–7.41)
Any incarcerations for father when child aged 1 to <5 years	25	*3.8*	2220	*1.3*	3.50	(2.35–5.22)
Any incarcerations for father when child aged 5 to <10 years	25	*3.8*	2314	*1.3*	3.47	(2.32–5.17)
Family functioning						
Child protection contact before child aged 10 years: Child or sibling victim of ⩾1 substantiated abuse	45	*6.9*	3395	*2.0*	4.29	(3.16–5.81)
Child or sibling subject of ⩾1 unsubstantiated notification, no substantiated cases	61	*9.3*	8052	*4.7*	2.59	(1.98–3.37)
Neither child nor siblings subject of any notifications	540	*83.8*	166 931	*93.3*	ref	–
Any parental corrective services contact before child aged 10 years	92	*14.0*	14 305	*8.3*	2.48	(1.99–3.10)
Family structure						
Mother age <20 years at child's birth	67	*10.2*	8432	*4.9*	2.11	(1.64–2.71)
Father age <25 or ⩾45 years at child's birth	191	*29.1*	30 292	*17.7*	1.82	(1.54–2.15)
Child's birth order 4th or greater	81	*12.3*	15 445	*9.0*	1.43	(1.13–1.80)
Family size at child's 10th birthday: One, three or four children	383	*58.4*	93 188	*54.3*	1.39	(1.16–1.65)
Two children	183	*27.9*	65 779	*38.3*	1.00	Reference
Five or more children	90	*13.7*	12 621	*7.4*	2.50	(1.94–3.22)
Mother not partnered at time of child's birth	105	*16.0*	13 773	*8.0*	2.21	(1.80–2.73)
Area-level socioeconomic/demographic environment^a^						
Value of index of socioeconomic disadvantage in lowest quintile	180	*27*.*4*	32 506	*19*.*0*	1.71	(1.44–2.04)
Remoteness classification: remote, very remote or unknown	99	*15.1*	19 168	*11.2*	1.12	(1.01–1.25)
Percentage of persons of Aboriginal or Torres Strait Islander descent ⩾25%	31	*4.7*	3749	*2.2*	1.52	(0.99–2.32)
Percentage of persons Australian born ⩽75%	480	*73.2*	124 111	*72.3*	1.17	(0.97–1.41)
Percentage of persons never married ⩾50%	47	*7*.*2*	5883	*3*.*4*	1.72	(1.27–2.34)
Percentage of one parent families ⩾20%	216	*32*.*9*	45 691	*27*.*0*	1.20	(1.01–1.43)
Unemployment rate ⩾5%	527	*80.3*	133 603	*77.9*	1.13	(0.92–1.39)
Percentage of persons living same residence as 5 years prior <40%	167	*25.5*	38 719	*22.6*	1.20	(1.00–1.45)
Percentage of persons living same residence as 1 year prior <70%	169	*25.8*	34 031	*19.8*	1.23	(1.02–1.49)
Percentage of persons living in semi-detached residences ⩾10%	191	*29.1*	40 291	*23.5*	1.19	(1.00–1.41)
Percentage of persons living in residences that are flats ⩾5%	154	*23*.*5*	30 222	*18*.*0*	1.27	(1.05–1.52)
Percentage of persons living in rented residences ⩾30%	251	*38.3*	50 025	*29.2*	1.25	(1.05–1.50)
Percentage of persons who do not speak English well between 1% and 2%	105	*16*.*0*	21 898	*13*.*0*	1.31	(1.07–1.62)
Total crime – arrest rate in 2002 ⩾50/1000 persons	642	*97.9*	167 025	*97.3*	1.83	(0.97–3.46)
Has high ethnic heterogeneity	90	*13*.*7*	13 701	*8*.*0*	1.39	(1.06–1.82)
Has more than minimal level of SEIFA inequality	597	*91.0*	154 715	*90.2*	1.16	(0.88–1.54)
Family-level sociodemographic status						
Father unknown/not registered at birth	50	*7.6*	5834	*3.4*	2.34	(1.75–3.12)
Father in lower skill occupation or unemployed at time of child's birth	138	*21.0*	26 710	*15.6*	1.62	(1.34–1.96)
Mother born in low income country or in Western Australia	432	*65.9*	101 431	*59.1*	1.35	(1.15–1.58)
Child of Aboriginal or Torres Strait Islander descent	128	*19.5*	12 167	*7.1*	3.38	(2.78–4.10)

Italics highlight that numbers so formatted are percentages.

a In area of mother's residence at child's birth, at 2001 census.

### Model development for five adversity domains

For each of the five adversity domains, the Cox model chosen to explain variation in schizophrenia diagnosis is summarised in Table 2. Adversity exposures for excluded influential observations can be found in online Supplementary Table S4, and changes in model parameter estimates due to their exclusion can be viewed in online Supplementary Table S5. Tests of proportionality assumptions did not show any substantial violations. Parameter estimates were considered robust to model specification after comparison with those corresponding to logistic regression formulation (online Supplementary Table S6).

**Table 2.** Risk of developing schizophrenia following exposure to adversity in each of five domains (hazard ratios and 95% confidence intervals)

Model M1 – Exposure to *discontinuity in parenting* and risk of schizophrenia	HR	95% CI
Mother hospitalised ⩾8 days when child aged 1 to <5 years	1.56	(1.32–1.84)
Mother hospitalised ⩾8 days when child aged 5 to <10 years	1.39	(1.13–1.70)
Child hospitalised ⩾8 days when child aged 15 days to <1 year	1.67	(1.18–2.37)
Child hospitalised ⩾8 days when child aged 1 to <5 years	1.41	(1.00–1.99)
Child hospitalised ⩾8 days when child aged 5 to <10 years	1.39	(0.86–2.23)
Father hospitalised ⩾8 days when child aged 1 -<5 years	1.43	(1.02–2.01)
Father hospitalised ⩾8 days when child aged 5 to <10 years	1.34	(0.97–1.87)
Any placement in foster care before child aged <10 years	4.70	(2.75–8.01)
Any incarcerations for father when child aged 1 to <5 years	1.80	(1.08–3.00)
Any incarcerations for father when child aged 5 to <10 years	1.56	(0.93–2.62)
Model M2 – Exposure to *family functioning* and risk of schizophrenia	HR	95% CI
Child protection contact before child aged 10 years: Child or sibling subject of ⩾1 unsubstantiated notification, no substantiated abuse	2.17	(1.64–2.86)
Child or sibling victim of ⩾1 substantiated abuse	3.51	(2.55–4.82)
No notification for any child or sibling within family	1.00	Reference
Any parental corrective services contact before child aged 10 years	1.80	(1.42–2.29)
Model M3 – Exposure to *family structure* and risk of schizophrenia	HR	95% CI
Father age <25 or ⩾45 years at child's birth	1.56	(1.29–1.88)
Family size at child's 10th birthday: One, three or four children	1.32	(1.11–1.58)
Two children	1.00	Reference
Five or more children	2.34	(1.82–3.02)
Mother not partnered at time of child's birth	1.66	(1.31–2.10)
Model M4 – Exposure to *area-level socioeconomic/demographic environment*^a^ and risk of schizophrenia	HR	95% CI
Value of index of socioeconomic disadvantage in lowest quintile	1.39	(1.13–1.70)
Percentage of persons never married ⩾50%	1.53	(1.12–2.11)
Percentage of one parent families ⩾20%	1.13	(0.95–1.35)
Percentage of persons living in residences that are flats ⩾5%	1.21	(1.00–1.46)
Percentage of persons who do not speak English well between 1% and 2%	1.31	(1.06–1.62)
Has high ethnic heterogeneity	1.40	(1.09–1.79)
Model M5 – Exposure to *family-level sociodemographic status* and risk of schizophrenia	HR	95% CI
Father unknown/not registered at birth	1.63	(1.20–2.23)
Father in lower skill occupation or unemployed at time of child's birth	1.36	(1.12–1.66)
Mother born in low-income country or in Western Australia	1.18	(1.00–1.39)
Child of Aboriginal or Torres Strait Islander descent	2.81	(2.27–3.47)

a In area of mother's residence at child's birth, at 2001 census.

The exposure profile of each child in the development sample was combined with the model parameters of Table 2 using Equation 1 to calculate each child's five continuous domain scores. Summaries of the distributions of these scores are presented in online Supplementary Fig. S1. The great majority of children were allocated scores in the lower ends of the distributions, with only small proportions allocated scores in the upper ranges.

### Global model development: combining domain models

Table 3 summarises the chosen global model which includes the five domain scores, together with interaction terms. This was chosen due to its combination of relative high concordance, good calibration and simplicity of application. Parameter estimates were considered robust (online Supplementary Table S6) and proportionality assumptions were considered valid.

**Table 3.** Risk of developing schizophrenia following exposure to adversity across five domains (hazard ratios and 95% confidence intervals)

Exposure to childhood adversity and risk of schizophrenia	HR^a^	95% CI
A1 Discontinuity in parenting	1.93	(1.52–2.45)
A2 Family functioning	1.90	(1.36–2.66)
A3 Family structure	1.66	(1.31–2.11)
A4 Area-level socioeconomic/demographic environment	1.99	(1.46–2.70)
A5 Family-level sociodemographic status	1.53	(1.26–1.86)
A1.A4 Interaction	0.58	(0.37–0.90)
A2.A3 Interaction	0.65	(0.45–0.93)
All other Aa.Ab interactions, a < b	0.00	na

a Exposure to adversity in each domain is measured on a continuous scale as a weighted sum of exposures defined in Table 2 and calculated using Equation 1. The hazard ratio of a domain is the estimated factor increase in the hazard for a 1 unit increase in score in that domain.

The global score – the EAS-Sz score – was calculated for each child in the development sample by applying Equation 2 to the model parameters of Table 3. A distributional summary of these scores is contained in online Supplementary Fig. S1.

### Model validation: calibration

**Fig. 1.**
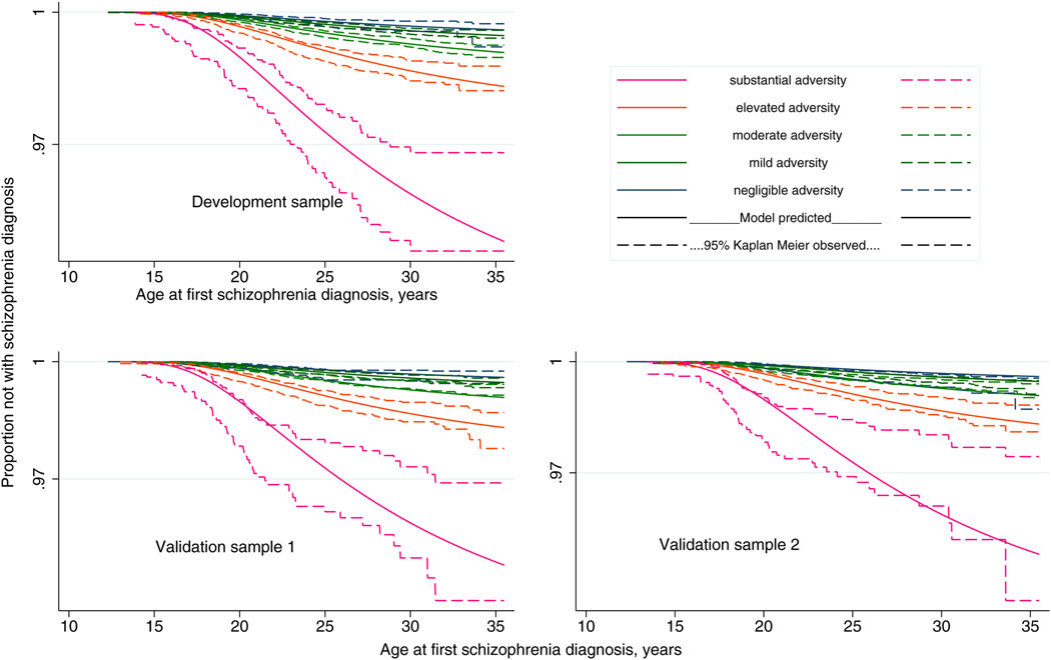
Calibration illustration for five prognostic risk strata of exposure to childhood adversity. Observed 95% limits for Kaplan–Meier curves and predicted time to diagnosis curves.

Five risk stratification categories were defined in the development sample, representing increasing levels of exposure to adversity: (i) negligible: 0 ⩽ EAS-Sz < 0.1 [*N* = 20 304, with Sz diagnosis = 33 (0.16%)]; (ii) mild: 0.1 ⩽ EAS-Sz < 0.5 [*N* = 75 815, with Sz diagnosis = 179 (0.24%)]; (iii) moderate: 0.5 ⩽ EAS-Sz < 1.0 [*N* = 52 639, with Sz diagnosis = 229 (0.44%)]; (iv) elevated: 1.0 ⩽ EAS-Sz < 2 [*N* = 19 889, with Sz diagnosis = 152 (0.76%)]; and (v) substantial: EAS-Sz ⩾ 2.0 [*N* = 2941, with Sz diagnosis = 63 (2.14%)]. The corresponding distributions for validation sets 1 and 2 are presented in Table 4 and display dose response. The proportion of children in validation sets 1 and 2 who received a schizophrenia diagnosis before the end of their follow-up increased from 0.19% and 0.26% respectively for those with EAS-Sz defined ‘negligible’ risk to 2.19% and 1.85% respectively for those with ‘substantial’ risk. In Fig. 1, the *model assumed* average predicted time-to-diagnosis curve for all children in each of the risk categories is presented, together with 95% CI limits of the observed Kaplan–Meier time-to-diagnosis curves. The illustration is repeated for the development sample and each validation sample. The observed Kaplan–Meier curves displayed consistency with model predictions.

**Table 4.** Validation of Cox models describing risk of schizophrenia following exposure to adversity (distribution of schizophrenia diagnoses across risk categories in validation samples for global model and Harrell's Concordance statistics for global and domain models)

	Validation set							
Model describing risk of schizophrenia following exposure to adversity	Set 1				Set 2			
	Full sample		Children with Sz diagnosis		Full sample		Children with Sz diagnosis	
**Global model** – exposure across five domains, as defined in Table 3	*N*	% Sample	*N*	% Category	*N*	% Sample	*N*	% Category
Risk category negligible: 0.0 ⩽ EAS-Sz < 0.1	15 258	*11*.*9*	29	*0*.*19*	15 464	*12*.*0*	40	*0*.*26*
Mild: 0.1 ⩽ EAS-Sz < 0.5	56 781	*44*.*1*	134	*0*.*24*	56 664	*44*.*0*	158	*0*.*28*
Moderate: 0.5 ⩽ EAS-Sz < 1.0	39 289	*30*.*5*	144	*0*.*39*	39 536	*30*.*7*	145	*0*.*37*
Elevated: 1.0 ⩽ EAS-Sz < 2.0	15 174	*11*.*8*	117	*0*.*77*	14 867	*11*.*6*	109	*0*.*73*
Substantial: EAS-Sz ⩾ 2.0	2187	*1*.*7*	48	*2*.*19*	2159	*1*.*7*	40	*1*.*85*
Harrell's Concordance	0.655				0.624			
**Single domain models**, as defined in Table 2								
Harrell's Concordance								
M1 – Discontinuity in parenting	0.597				0.589			
M2 – Family functioning	0.597				0.583			
M3 – Family structure	0.609				0.591			
M4 – Area-level socioeconomic/demographic environment	0.579				0.551			
M5 – Family-level sociodemographic status	0.598				0.575			

Italics highlight that numbers so formatted are percentages.

### Model validation: discrimination

Predictive performance for selected models is summarised in Table 4. The global model produced Harrell's Concordance values of 0.656 and 0.624 for validation sets 1 and 2 respectively.

For models describing individual domains, the highest values of Harrell's Concordance were for A3 Family structure (0.609 in validation set 1 and 0.591 in validation set 2).

## Discussion

### Principal findings

To the best of our knowledge, we have derived and validated the first comprehensive early adversity scale for schizophrenia [EAS-Sz] describing the risk of schizophrenia diagnosis after exposure to broadly defined childhood adversity, using routinely (and prospectively) collected register data. The scale alone accurately predicts schizophrenia above chance, and so has good potential to be combined with other risk factors for schizophrenia as a tool to help untangle the relative contributions of genetic and environmental liability.

Our method estimated the relative impact of various components of adversity exposure, simultaneously adjusting for other exposures. The scale components, all derived from electronic register data, cover a wide range of exposures across multiple broad domains up to a child's 10th birthday and are far greater in number than any sets of exposures previously investigated. Using a large population cohort, we developed the scale using a partitioned 40% sample and evaluated its internal validity using two separate 30% samples. The main scale was a complex global scale which incorporated five domains: (i) discontinuity in parenting, (ii) family functioning, (iii) family structure, (iv) area-level socioeconomic/demographic environment and (v) family-level sociodemographic status, and its values of Harrell's Concordance were well above chance, ranging from 0.624 to 0.656. Although superficially modest, we highlight that these concordance values are achieved for a partial prediction model. We note that the score does not include any proxy measures for genetic liability to schizophrenia (familial diagnosis history) or other risk factors that have been reported in the literature such as cognitive function, obstetric complications and cannabis use, and only includes exposures up to age 10 years. Predicted time-to-diagnosis curves for five risk strata calculated from the development sample were consistent with 95% CI bounds of the corresponding observed Kaplan–Meier curves in both validation samples. In addition to the global scale, we produced five subscales, each based on a single domain of adversity. These also indicated prediction above chance (Harrell's Concordance values range 0.551–0.609). These results indicate that, while some predictive capacity can be achieved from examining adversity exposure in a single domain, more can be gained by considering a broad measure of adversity across many domains. We consider this a particular strength of our scale.

### Strengths and limitations

Although simple in application, our scale is unique in the level of sophistication of data used in its derivation, drawing on high-quality State electronic registers of excellent coverage for a large population. The information was prospectively collected, avoiding recall bias. In addition, by accessing data retrospectively, we covered a large time span efficiently. Importantly, the scale allows for different severity weightings for different exposures, some differing by age at occurrence. Finally, by limiting exposure time from birth to a child's 10th birthday, well before the outcome of schizophrenia diagnosis (in all but the rarest of cases), we have avoided potential confounding with premorbid behaviours associated with attenuated or at-risk mental states in early psychosis.

Of great statistical advantage, this scale allows researchers to incorporate many measures of a complex adversity profile into their analyses as a single metric – leaving degrees of freedom free to estimate other contributions to risk of schizophrenia such as genetic liability. See, for example, Vargas and Mittal (2018). As its derivation was achieved through the application of straightforward statistical techniques, other researchers can emulate the process.

The validation of our scale using large datasets which were randomly selected from our cohort gives confidence to the scale's ability to accurately represent risk of schizophrenia in Western Australia, and augers well for its generalisability to other similar populations. However, we believe further validation of EAS-Sz by testing its application with registers from other jurisdictions would confirm whether the relationship between early childhood adversity exposure and schizophrenia outcome that we have quantified holds true when applied to electronic register records elsewhere in the world. This remains an important future step.

Our results have not yet been assessed for specificity to schizophrenia compared to other mental health outcomes, many of which have also been associated with exposure to early adversity.

Our measures of adversity exposure used in constructing EAS-Sz have been derived from records in State registers. We acknowledge that some measures of exposure may not always correspond to an expected increased risk of an undesirable outcome at an individual level and can reasonably be labelled ‘blunt’ measures. However, the substantial difference in prevalence of schizophrenia diagnosis between children classified with ‘substantial’ risk compared to lower risk levels gives support to the ability of EAS-Sz to identify childhood adversity at levels of severity that have greatest impact on the risk of schizophrenia diagnosis.

Despite the breadth of our information, some important exposures could not be considered because they are not recorded in registers, for example, experience of bullying, or family rearing style. Also we did not have access to information for children of mothers on the psychiatric register with psychiatric disorders other than psychotic illness. This was consistent with the ethics approval for the original e-Cohort design; however, we acknowledge that the omission of these children could exclude some patterns in adversity exposure and subsequent schizophrenia risk. In addition, follow-up time for those children born late in the study period was short and, due to their age, they had not passed through the risk period for developing schizophrenia.

Despite minor indications that the model predicted rates of diagnosis at the lower end of the observed 95% Kaplan–Meier estimates for younger ages and towards the higher end of those observed for older ages, we observe that our calibration is generally very good.

Our Department of Child Protection data covering abuse notifications and any subsequent substantiations only include records from 1989 onwards, which reduces the data coverage for around 40% of the children in our cohort. We estimate this translates to an undercount of approximately 20% of children exposed to a substantiated allegation of child abuse in their maternal family (i.e. we estimate we have missed identifying 0.4% of the cohort population as being exposed to a substantiated allegation).

### Results in relation to previous studies

There are few reports on the validation and performance of adversity scales. The data-driven method adopted by Pries et al. (2019) to develop their Exposome Score is one of the closest in approach to ours, although major differences remain: they developed their predictive model using far fewer exposure measures, most of which were determined by recall, and in the case of patients, after diagnosis. In general, their predictive exposures were more chronologically concurrent with outcome than our own, which we deliberately limited to exposures before 10 years of age. Due to the observational structure of their training sample, just under 40% had a diagnosis, time of follow-up was not relevant, and due to the age of the participants (mean = 34 years, s.d. = 10), the likelihood of censored outcome in the controls could be assumed non-existent. The predictive model they developed using logistic regression was found to have an area under the receiver operating curve (AU-ROC) of 0.73 when tested in a separate observational sample (Pries et al., 2019). Pries et al. further validated their Exposome Score, reporting an AU-ROC of 0.84 after application to a third cross-sectional sample (Pries et al., 2020).

In comparison, we measured baseline exposure, prospectively recorded from objective registers, at 10 years old, and incorporated variable follow-up time (mean age 24 years, range 10–35.5 years) with high levels of censoring using Cox regression. For those who developed schizophrenia, the mean age at diagnosis was 22 years. We report Harrell's Concordance (the AU-ROC equivalent for Cox regression) of 0.656 and 0.624 for our two internal validation samples.

### Conclusions and future directions

The causal pathway to schizophrenia is complex. Environmental factors have long been implicated in schizophrenia risk, but much remains to be understood about the role of these factors in schizophrenia liability (van Os et al., 2010). Capturing these environmental risks adequately and in a replicable form has been a fraught exercise (Guloksuz, van Os, & Rutten, 2018). However, perseverance in developing more comprehensive measures of multidimensional environmental exposures is critical: as Vassos et al. (2020) highlight, quantifying cumulative environmental risk is vital, given its potential with respect to intervention and prevention. Our scale offers an objective method of efficiently combining multiple exposures to adversity that, in the literature, have been associated with risk of schizophrenia. The method affords different weightings to different exposures and considers potential interactions, to create a comprehensive global metric of cumulative risk that also includes an option for parsing adversity risk into its underlying sub-domains. To further increase the portability of the scale across platforms and research groups, future developments may include remodelling it as a nominal scale, where each increment in risk is characterised by a smaller set of necessary and sufficient exposures. In developing the nominal scale, approximations with differing levels of detail would be assessed in terms of the relative loss in predictive capacity incurred, against the benefit of improved simplicity of risk measurement calculation.

Our scale provides a nuanced tool for furthering our understanding of the role of cumulative adversity in schizophrenia risk, including its interplay with genetic liability and other exposures such as cognitive impairment and obstetric complications. In current work we are studying these interplays with the global EAS-Sz score and separate domain-level scores, including the specificity of EAS-Sz and its components to schizophrenia, in contrast with other mental health outcomes. Of great importance, its application to whole-population registers allows for retrospective analysis of data from large cohorts to identify modifiable factors along the developmental pathway to schizophrenia that may also influence genetic vulnerability and become potential targets for intervention and prevention.

## Supplementary material

For supplementary material accompanying this paper visit https://doi.org/10.1017/S0033291722001945. click here to view supplementary material
