# Supplementary material for: Development and initial validation of a multivariable predictive Early Adversity Scale for Schizophrenia (EAS-Sz) using register data to quantify environmental risk for adult schizophrenia diagnosis after childhood exposure to adversity
Source: Psychol Med. 2022 Jul 12;53(11):4990–5000. doi: 10.1017/S0033291722001945 (PMC10476059; doi:10.1017/S0033291722001945)
Supplement: Supplementary file 1 [file S0033291722001945sup001.docx]

# Supplementary materials

**Covariate dictionary**. Detailed descriptions of adversity exposure measures and data sources

**Supplementary Table S1.** Data coverage for constructed adversity exposures. Development sample.

**Supplementary Table S2**. Adversity exposures for children in development sample excluded or censored due to death. Counts and percentages.

**Supplementary Table S3**. Exposure to adversity in validation samples (Number and percentage).

**Supplementary Table S4**. Alternative model parameterisations for risk of developing schizophrenia following exposure to adversity across domains A1 – Discontinuity in parenting and A4 – Area level socio-economic/demographic environment. Hazard ratios and 95% Confidence intervals.

**Supplementary Table S5**. Observations excluded from final model parameter estimation due to large influence. N=27 observations, all with schizophrenia diagnosis. Adversity exposure comparison with non-excluded observations.

**Supplementary Table S6**. Parameter estimates derived from development sample including observations with large influence. Comparison with optimum models

**Supplementary Table S7**. Elementary validations for models. Robustness of estimated Cox model hazard ratios - comparison with estimated logistic regression odds ratios (Ratios and 95% Confidence intervals). Proportional hazards validity – test for non-zero correlation of Schoenfeld residuals with time (p values).

**Supplementary Table S8.** Risk of developing schizophrenia following exposure to adversity across five domains. Alternative Global models. Hazard ratios and 95%Confidence intervals.

**Supplementary Table S9**. Alternative Global Cox model parameterisations describing risk of schizophrenia following exposure to adversity. Harrell’s Concordance statistics.

**Supplementary Figure S1**. Distributions of predicted log hazard ratios for five domain models and Global model, derived from development sample.

## Covariate dictionary

### Covariates in manuscript

(*See also section 3. which describes the larger pool of candidate variables from which the final variables were chosen*).

A1 - Discontinuity in parenting before the age of 10 years covered any separation of a child from a parent categorised by the child’s developmental epoch: i) under one year of age; ii) ages one to four; and iii) ages five to nine.

- *Hospitalisations* of the *child*, the *mother* or the *father* (sourced from the **Hospital Morbidity Data Collection**) were classified as adverse if multiple admissions which totalled more than 8 days occurred within in any developmental epoch. To exclude a child’s birth related hospital stay being included as separations between mother and child, the less than 1 year period was defined to begin at age 15 days for the child and their mother, rather than 0 days as for the fathers.
- P*arental deaths* before a child’s 10^th^ birthday were sourced from the **Western Australian Death Registry**.
- The **Department of Communities, Child Protection and Family Support Division database** provided information pertaining to any child *placement in foster care* before their 10th birthday*.*
- Custodial sentences leading to *incarceration* of a child’s mother or father during each of a child’s developmental epochs were determined from the **Western Australian Department of Justice**.

A2 - Family functioning

- *Corrective services contacts* for a child’s mother or father, before the child’s 10th birthday, were obtained from the **Western Australian Department of Justice**. As well as incarceration, these contacts include diversionary programmes and non-custodial orders.
- *Child protection contacts* before a child’s 10^th^ birthday covering any notification of allegations of child abuse (sexual, physical and emotional) and neglect were sourced from the **Department of Communities, Child Protection and Family Support Division database** and were classified as adverse at two levels i) if, before a child’s 10th birthday, any notification of abuse was made related to a child or any of their maternal siblings but with none of those notifications substantiated and ii) as for i), but with at least one notification of abuse being substantiated.

A3 - Family structure

- *mother’s age* at time of child’s birth was calculated using the **Midwives’ Notification System**, and categorised as less than 20 years or at least 20 years.
- *paternal age* at child’s birth was calculated for those fathers with valid **Birth Registration** details and categorised as less than 25 years, at least 25 years but less than 45 years, at least 45 years.
- *marital status* (partnered / unpartnered) of the child’s mother at the time of a child’s birth and the
- child’s *birth order* (1st, 2nd or 3rd compared to 4th or greater) amongst maternal siblings were determined from the **Midwives’ Notification System**.
- *size* of the *family* (2 children, 5 or more children, or 1, 3 or 4 children) a child belonged to at the time of their 10^th^ birthday was calculated by combining birth order with any **Hospital Morbidity Data Collection** entries after 2001 which identified further live births to a child’s mother.

A4 - Area level socio-economic/demographic environment

The Australia Bureau of Statistics uses a hierarchical geographic classification system {Australian Bureau of Statistics, 2006 #49} for collection and publication of official statistics. The finest level at which statistics were published for the 2001 Census was the Census Collection District (CCD), a grouping of approximately 200 geographically proximate households. The CCD corresponding to a mother’s address at the time of the child’s birth was provided by the Data Linkage Branch which was matched to the relevant area level 2001 census statistics. In a minority of cases where CCD level statistics were not available, aggregation at postcode level CCD was used.

- *Socioeconomic status* was determined using the **Australian Bureau of Statistics Socio-Economic Indexes for Areas** {Australian Bureau of Statistics, 2008 #48}. We used the Index of Relative Socioeconomic Disadvantage, comparing those children born in areas of the quintile of greatest disadvantage to all other children. The index used was that for the available census (1986, 1991, 1996 or 2001) closest in time to the date of birth. Transformation of index values to quintiles allowed comparison across subject birth years.
- *Remoteness or level of urbanicity* of the geographical area of a mother’s residence at the time of her child’s birth was determined using the **Australian Standard Geographic Classification—Remoteness Area**, as defined in 2001. {Australian Bureau of Statistics, 2006 #49}. We compared those children born to mothers resident in Remote or Very Remote areas to children born to mothers resident in all other areas.

Further area level measures determined from 2001 census information were percentage of persons in a CCD who:

- identified as *Indigenous* (of Aboriginal or Torres Strait Islander descent) ( <25% or >=25%)
- were *Australian born* (>=75% or <75%)
- *never married* (<50% or >=50%)
- lived in *one parent families* (<20% or >=20%)
- were *unemployed* (<5% or >=5%)
- lived in different *residence to 5 years* previous (<60% or >=60%)
- lived in different *residence to 1 year* previous *(<30% or >=30%)*
- lived in *semi-detached* dwellings *(<10% or >=10%)*
- lived in *flats* *(<5% or >=5%)*
- lived in *rented* dwellings *(<30% or >=30%)*
- had *no post school* *qualifications* (<40% or >=40%)
- *spoke a language other than English at home and did not speak English well* (>=2% or <2%).
- *Ethnic heterogeneity* was calculated for each CCD using the formula: E =1-$P_{1}^{2}-P_{2}^{2}$ where P_1_ is the percentage of residents in that CCD who identified as Indigenous, and P_2_ is the percentage of residents in that CCD who spoke a language other than English at home and who did not speak English well.{Morgan, 2008 #149} This measure is at its lowest when the diversity within a population is highest. Areas of high heterogeneity were defined as those where **E** was calculated to be less than 0.99.
- *Total area-level crime* was based on the arrest rate per 1000 residents in a Local Government Area during 2002, as provided by the **Western Australian Crime Research Centre.** Children born in areas where at least 50 arrests per 1000 residents were recorded were compared to all other children.
- *socioeconomic inequality* for a postcode was derived from the standard deviation of the distribution of the **Index of Relative Socioeconomic Disadvantage** scores for CCDs within that postcode. Across all postcodes, the distribution of standard deviations was categorised with the quartile of smallest standard deviations defined as minimal localised inequality.

A5 - Family level sociodemographic status

- A binary variable was created to indicate whether the child’s *known father* had been named on the State **Birth Registration** record.
- The *child’s Indigenous* status was scored positive if the child and/or either parent was identified as Indigenous (of Aboriginal or Torres Strait Islander descent) in any of the data sources available.
- Maternal and paternal place of birth were extracted from State **Birth Registration** records. In turn, *Migrant status* of parents was classified according to the affluence of the country of origin, using **World Bank Income Categories**, and for Australian born parents, whether they had been born within or outside Western Australia. Based on patterns of response in preliminary models, children of Western Australian born parents were grouped with children of parents born in lower income countries
- The State **Birth Registration’s** record of a *father’s* *occupation* at the time of a child’s birth was classified according to skill level based on the **Australian Bureau of Statistics Occupation Classifications.** Categorisation was defined as fathers in employment requiring middle to high skill level compared to all other employment statuses such as low skill, unemployed, not in labour-force etc).

### Descriptions of Data sources

**Hospital Morbidity Data Collection**

Records available 1^st^ January 1980 to 31^st^ December 2017

The collection is managed by the WA Health System and records all episodes of care that occur in public and licensed private acute and psychiatric hospitals as well as licensed private day surgeries. Excluded episodes of care include those pertaining to patients attending emergency, outpatient or community health services, those in residential aged care facilities, still births and mothers electing to deliver at home

**Mental Health Information System**

Records available 1^st^ July 1966 to 31^st^ December 2015.

Covers records of all public and private inpatient hospital admissions, as well as public outpatient and ambulatory care contacts with mental health services across the State.

**Western Australian Death Registry**

Records available 1^st^ January 1980 to 19^th^July 2018 for all deaths registered in Western Australia

**Department of Communities, Child Protection and Family Support Division database**

Records available 1^st^ July 1989 to 29^th^ February 2016 for notifications.

Records available 1^st^ April 1981 to 12^th^ May 2016 for placements in care.

The Department of Communities, Child Protection and Family Support Division record all notifications of child abuse (sexual, physical and emotional) and neglect meeting standard Australian definitions {Bromfield, 2008 #576;Bromfield, 2008 #576} and the outcome of subsequent investigations – whether substantiated or not. The date of reporting does not always reflect the date of abuse, as reporting of the abuse may happen years after the event/s. The Division also records all placements of children in foster care and the duration of such placements.

**Western Australian Department of Justice**

Records available 1^st^ January 1980 to 15^th^ October 2011.

The Western Australian Department of Justice holds electronic records dating back to the early 1970s. The data include information on youth detention, adult prison populations and non-custodial orders (adult community-based and youth justice orders). They cover Western Australia’s 14 public prisons, two privately run prisons, five work camps and one detention centre, as well as offenders on probation, parole, other community orders and diversionary programmes for young people. The minimum age for inclusion on the Western Australian corrective services records is 10 years of age.

**Midwives Notification System**

Records available 1st January 1980 to 31st December 2001.

The Midwives Notification system {Gee, 1994 #37} includes mandatory, prospectively collected data on all infants born in Western Australia at 20 weeks gestation or more, or weighing at least 400 grams, including home births, and not restricted to live births. Demographic information for the mother includes age, date of birth, marital status, postcode of residence and ethnic identity. (Outcomes of previous pregnancies, occurrence of any complications, medical conditions or treatments during pregnancy and details of labour including any complications or interventions are also recorded). Vital details pertaining to the baby include date and time of birth, gender, whether baby was one of a multiple birth event and if baby was stillborn or alive.

**Birth Registration records – mother and father details**

Records available for births 1st January 1980 to 31st December 2001.

In Western Australia, a Birth Registration Form must be lodged with the Registry of Births Deaths and Marriages within 60 days of the birth of a child to enable the newborn to be officially recognised for a range of services. This must be completed and signed by both parents. If only one parent has signed, they must provide an explanation why the other parent has not signed. Information requested of each parent includes Indigenous identity, age, date and place of birth, and occupation.

**Australian Bureau of Statistics Socio-Economic Indexes for Areas (Index of Relative Socioeconomic Disadvantage)**

The Socio-economic Indexes for Areas (SEIFA) is a set of indexes created by the Australian Bureau of Statistics from census data. For general information about all SEIFA, including SEIFA_A = Index of Relative Socio-economic Disadvantage, see [*https://www.abs.gov.au/ausstats/abs@.nsf/mf/2039.0*](https://www.abs.gov.au/ausstats/abs@.nsf/mf/2039.0) .

SEIFA_A is constructed differently for each Census, however this study used quintiles of SEIFA_A to assign comparable values for the SEIFA_A index for each census year.
 Census Birth years

1986 1980 – 1987

1991 1988 – 1993

1996 1994 – 1998

**Australian Standard Geographic Classification—Remoteness Area**

Remoteness Area categories are based on the Accessibility/Remoteness Index of Australia (ARIA) maintained by the National Centre for Social Applications of GIS (GISCA) at the University of Adelaide. Its calculation includes consideration of the road distance of a location from the nearest population centres providing access to goods and services, taking into account population size. Since the 2001 census, the Australian Bureau of Statistics (ABS) has defined a ‘Remoteness Area classification Index (RA)’ for each non-overlapping Census Collection District (CD), based on the methodology of the ARIA. Each Collection District is then categorised, according to the value of the Index as one of 5 levels of urbanicity: 'Major Cities of Australia', 'Inner Regional Australia', 'Outer Regional Australia', 'Remote Australia', and 'Very Remote Australia'.

The ABS RA measure is superior to a postcode-only based definition of urbanicity due to increased geographical precision and rigor of definition. A limitation of using the RA classification correct at Census 2001 is the omission of historical RA trends. For example, the residence of the mother of a child born in 1980 may have been considered Inner Regional at this time. With increased urban spread, the very same residence may be in a Major Cities area in 2001. The allocation of the 2001 RA value to the residence of that child does not acknowledge that for at least part of the child’s early life, the residential environment was considered Inner Regional.

**Western Australian Crime Research Centre**

The Crime Research Centre of the University of Western Australia was established in 1989 as a major and innovative contributor to the collection and analysis of statistical data on crime in Western Australia. It published annual crime and justice statistics which enhanced the understanding of the extent of the crime problem in the state and trends in criminal justice. The arrest rate per 1,000 residents in a Local Government Area during 2002 were provided to the authors for the specific requirements of this study.

**World Bank Income Category rankings**

The World Bank Income Category list of global economies (July 2012) was accessed 5^th^ March 2013. <http://siteresources.worldbank.org/DATASTATISTICS/Resources/CLASS.XLS>. Based on Gross National Income figures for 2011, each economy was classified as Low Income, Lower middle income, Upper middle income, or High income. The Standard Australian Classification of Countries (SACC) - Australian Bureau of Statistics was used to link countries to economies. <http://www.abs.gov.au/ausstats/abs@.nsf/Latestproducts/3E3848A2DCF7FF4FCA25744B001535DB?opendocument>

**Australian Bureau of Statistics occupation classifications**

The Australian Bureau of Statistics publication, *1220.0 - Australian Standard Classification of Occupations (ASCO) Second Edition, 1997* classifies 986 unique occupations into 9 Major Groups which map to 5 ordinal Skill Levels.

<http://www.ausstats.abs.gov.au/ausstats/free.nsf/0/A86A0162E6F672DFCA256ADB001D10D4/$File/asco.pdf>

### 3. Raw constructs considered in preliminary screening for association with schizophrenia.

Before arriving at the covariates referenced in section 1, we considered a wider range of adversity exposures that were considered to have potential to measurably impact the risk of schizophrenia diagnosis. Where the register data provided sufficient scope and accuracy for their calculation, we used a pragmatic, data-driven approach, to construct categorical variables. Categories were designed to retain maximum information about the relationship between the candidate variable and schizophrenia risk, while summarising the relationship parsimoniously and providing for the possible creation of equivalent variables by other researchers. Thus, while we acknowledge that information can be lost by categorising a continuous measure, we anticipated that linear relationships would be insufficient to account for likely complex associations between measures of adversity and schizophrenia. Relationships were initially assessed with several categories so that general functional forms could be identified from the data (rather than assumed prior) and then piecewise functions could be constructed to approximate the functional forms. In summary, we assessed bivariate associations between categorical variables and schizophrenia (specifically Nelson-Aalen graphs and hazard ratios) to i) exclude those variables with insufficient evidence of association from further consideration and ii) for those variables with evidence of association, to identify where categories could be collapsed without substantial loss of explanation of varying risk. The full range of variables considered, from which final manuscript variables were derived, are listed below.

A1 - Discontinuity in parenting before the age of 10 years covered any separation of a child from a parent categorised by the child’s developmental epoch: (i) under one year of age; (ii) ages one to four; and (iii) ages five to nine.

- Numbers of, and length of *Hospitalisations* of the child, the mother or the father (sourced from the Hospital Morbidity Data Collection) were classified as: no recorded hospitalisations, single day procedure/s only, single admission of any length of at least overnight, multiple admissions totalling 1-7 nights, multiple admissions totalling at least 8 nights. To exclude a child’s birth related hospital stay being included as separations between mother and child, the less than 1 year period was defined to begin at age 15 days for the child and their mother, rather than 0 days as for the fathers.
- P*arental deaths* were sourced from the Western Australian Death Registry separately for mothers and fathers and, depending on date of death, attributed to the corresponding epoch of the child’s development.
- The Department of Communities, Child Protection and Family Support Division database was searched for any child *placement in foster care.* Numbers of and length of placements were recorded for each of a child’s developmental epochs*.*
- Custodial sentences leading to *incarceration* of a child’s mother or father (number of and durations) were determined from the Department of Justice records. Tallies were considered separately for each epoch of the child’s development.

A2 - Family functioning

The Department of Communities, Child Protection and Family Support Division record notifications of allegations of child abuse (sexual, physical and emotional) and neglect meeting standard Australian definitions {Bromfield, 2008 #576; Bromfield, 2008 #576} and the outcome of subsequent investigations – whether substantiated or not. It should be noted that the date of notification can occur well after the date the alleged abuse took place, and, due to the nature of the trauma surrounding the allegations, the accuracy of the dates of alleged offences is hard to establish. Hence, our inclusion of only notifications received up to a child’s 10^th^ birthday will necessarily exclude some instances of abuse which had occurred but had not yet been reported. Recognising the possibility of trauma by association, we considered any occurrences of child protection contact relating to a child’s maternal siblings as well as those relating specifically to the child.

- The number of *Child protection contacts* before a child’s 10^th^ birthday were classified as: no allegations made relating to child or any of their maternal siblings, number of allegations relating to any sibling, number of substantiated allegations related to any sibling, number of allegations related to child, number of substantiated allegations related to child.

Criminal offending data were obtained from the Western Australian Department of Justice and, as well as detention, include information on diversionary programmes and non-custodial orders.

- The number of any such corrective services contacts for a child’s mother or father, before the child’s 10th birthday, were noted.

A3 - Family Structure

Using the Midwives’ Notification System,

- *mother’s age* at time of child’s birth was calculated and categorised as less than 20 years, 20-24 years, 25-29 years, 30-34 years or at least 35 years.
- Maternal *marital status* (never married, widowed, divorced, separated, married (including defacto), other status) at the time of a child’s birth was extracted,
- both the child’s *birth order* amongst maternal siblings and
- whether they were part of a *multiple birth* event

were determined. This data, combined with any Hospital Morbidity Data Collection entries after 2001 which identified further live births to a child’s mother

- allowed calculation of the *size of the family* a child belonged to at the time of their 10th birthday.
- For those fathers with valid birth registration details, *paternal age* at child’s birth was calculated and categorised as for mothers with further refinement for 35-39 years, 40-44 years and at least 45 years.

A4 - Area level socio-economic/demographic environment

The Australia Bureau of Statistics uses a hierarchical geographic classification system {Australian Bureau of Statistics, 2006 #49} for collection and publication of official statistics. The finest level at which statistics were published for the 2001 Census was the Census Collection District (CCD), a grouping of approximately 200 geographically proximate households. The CCD corresponding to a mother’s address at the time of the child’s birth was provided by the Data Linkage Branch which allowed access to relevant area level 2001 census statistics. In a minority of cases where CCD level statistics were not available, aggregation at postcode level CCD was used.

- *Socioeconomic status* was determined using the Australian Bureau of Statistics Socio-Economic Indexes For Areas {Australian Bureau of Statistics, 2008 #48}. We used the Index of Relative Socioeconomic Disadvantage. The index used was that for the available census (1986, 1991, 1996 or 2001) closest in time to the date of birth. Transformation of index values to quintiles allowed comparison across subject birth years.
- The geographical *remoteness or level of urbanicity* of the mother’s residence at time of a child’s birth was determined using the Australian Standard Geographic Classification—Remoteness Area, as defined in 2001. {Australian Bureau of Statistics, 2006 #49}. Remoteness Area categories are based on the road distance of a location from the nearest population centres providing access to goods and services, taking into account population size. They are classified as: Major City, Inner Regional, Outer Regional, Remote and Very Remote.

It is noted that both Socioeconomic status and Remoteness are composite measures, which may intrinsically incorporate some information measured by the following additional area level measures determined from 2001 Australian Bureau of Statistics Census Data. As such, additional census data variables were assessed for association with schizophrenia after adjustment for both Socioeconomic status and Remoteness. Alternative distributional categorisations were considered: i) approximately 3 categories corresponding to natural cut points of the relevant distribution, and ii) quintiles for each of the variables ’percentage of persons in a CCD who: '

- identified as *Indigenous* (of Aboriginal or Torres Strait Islander descent), were *Australian born*, *never married*, lived in *one parent families,* were *unemployed*, lived in the *same residence as 5 years* previous, lived in the *same residence as 1 year* previous, lived in *semi-detached* dwellings, lived in *flats* dwellings, lived in *rented* dwellings, had *no post school* *qualifications*, or *spoke a language other than English at home and did not speak English well*.
- *Ethnic heterogeneity* was calculated for each CCD using the formula: E =1-$P_{1}^{2}-P_{2}^{2}$P_1_ where P_1_ is the percentage of residents in that CCD who identified as Aboriginal or Torres Strait Islander, and P_2_ is the percentage of residents in that CCD who spoke a language other than English at home and who did not speak English well.{Morgan, 2008 #149} This measure is at its lowest when the diversity within a population is highest. Three heterogeneity categories were defined according to natural cut points in the distribution of E, i) lowest diversity: 0.999<=E<=1, ii) some diversity: 0.99<=E<0.999, and iii) greatest diversity: 0<=E<0.99.
- *Area-level crime total,* against *person* and *other* was based on the arrest rate per 1000 residents in a Local Government Area during 2002 and considered as both quintile and natural cut point categorisations of the distributions.
- For each postcode, the distribution of the Index of Relative Socioeconomic Disadvantage -Census 2001 scores (one score for each CCD within the postcode) was summarised by its standard distribution. Across all postcodes, the distribution of standard deviations was categorised into quartiles to provide a measure of localised *socioeconomic inequality.*

A5 - Family level sociodemographic status

- A binary variable was created to indicate whether the child’s *known father* had been named on the State Birth Registration record.
- The *child’s Indigenous* status was scored positive if the child and/or either parent was identified as Indigenous (of Aboriginal or Torres Strait Islander descent) in any of the data sources available.
- Maternal and paternal place of birth were extracted from State Birth Registration records. *Migrant status* of parents was classified according to the affluence of the country of origin, using World Bank Income categories , and for Australian born parents, whether they had been born within or outside Western Australia. Place of birth was then categorised as born in Western Australia, born elsewhere in australia, born overseas in a country of low income or born overseas in a country of high income
- The State Birth Registration’s record of a *father’s* *occupation* was classified according to skill level based on the Australian Bureau of Statistics (ABS) occupation classifications. The ABS uses a 5 skill level categorisation which we collapsed to 3 levels: Levels 1 and 2 into ‘Generally higher skilled’, Skill Levels 3 and 4 to ‘Mid-range skills’, and Skill Level 5 as ‘Generally lower skilled’, while retaining separate categories for ‘Not in Labour force’ and ‘Unable to be determined’. Father’s occupation status was missing for 3.7% of known fathers (6,186/165,754).

### References

**Australian Bureau of Statistics** (2006). Statistical Geography Volume 1 - Australian Standard Geographical Classification (ASGC), Jul 2006. Catalogue no. 1216.0. Australian Bureau of Statistics: Canberra.

**Australian Bureau of Statistics** (2008). Socio-Economic Indexes for Areas (SEIFA) - Technical Paper 2006. Catalogue no. 2039.0.55.001. Australian Bureau of Statistics: Canberra.

**Bromfield, L. & Holzer, P.** (2008). A National Approach for Child Protection Project Report. . Australian Institute of Family Studies. : Melbourne.

**Gee, V. & Dawes, V.** (1994). Validation Study of the Western Australian Midwives' Notification System 1992. Occasional Paper 59. p. 18. Health Department of Western Australia.

**Morgan, V. A., Morgan, F., Clare, J., Valuri, G., Woodman, R., Ferrante, A., Castle, D. & Jablensky, A.** (2008). *Schizophrenia and offending: area of residence and the impact of social disorganisation and urbanicity*. Australian Institute of Criminology.

## Supplementary Table S1 – Data coverage for constructed adversity exposures. Development sample.

| **Adversity exposure** | **Total development sample N=171,588** |
| --- | --- |
| **Discontinuity in Parenting** |  |
| Mother hospitalised >= 8 days when child aged 15 days -<1 year | Complete; some censoring if mother (n=42) deceased <1 yr |
| Mother hospitalised >= 8 days when child aged 1 -<5 years | Complete; some censoring if mother (n=42+208) deceased <5 yr |
| Mother hospitalised >= 8 days when child aged 5 years -<10 years | Complete; some censoring if mother (n=42+208+389) deceased <10 yr |
| Child hospitalised >= 8 days when child aged 15 days -<1 year | Complete; some censoring if child deceased < 1 yr |
| Child hospitalised >= 8 days when child aged 1 -<5years | Complete; some censoring if child deceased < 5 yr |
| Child hospitalised >= 8 days when child aged 5 years -<10 years | Complete; some censoring if child deceased < 10 yr |
| Father hospitalised >= 8 days when child aged 0 days -<1 year | Complete; some censoring if father (n=129) deceased < 1 yr |
| Father hospitalised >= 8 days when child aged 1 -<5 years | Complete; some censoring if father (n=129+499) deceased < 5 yr |
| Father hospitalised >= 8 days when child aged 5 years -<10 years | Complete; some censoring if father (n=129+499+813) deceased < 10 yr |
| Deceased parents, either or both, before child aged <10 years | Complete |
| Any placement in foster care before child aged <10 years | Records available FROM 1^st^ April 1981. Affected N=8,343^[[1]](#footnote-1)^ |
| Any incarcerations for mother when child aged <10 years | Records available UNTIL 15^th^ October 2011. Censored N=1,572  some censoring if mother deceased < 10 yr |
| Any incarcerations for father when child aged 0 days -<1 year | Complete; some censoring if father deceased < 1 yr |
| Any incarcerations for father when child aged 1 -<5 years | Complete; some censoring if father deceased < 5 yr |
| Any incarcerations for father when child aged 5 years -<10 years | Records available UNTIL 15^th^ October 2011. Censored N=1,572  or if father deceased < 10 yr |
| **Family Functioning** |  |
| Child protection contact before child aged 10 years:  Child or sibling victim of >=1 substantiated abuse | Records available FROM 1^st^ July 1989. Affected N=70,474^[[2]](#footnote-2)^ |
| Child or sibling subject of >=1 unsubstantiated notification, no substantiated cases |  |
| Neither child nor siblings subject of any notifications |  |
| Any parental corrective services contact before child aged 10 years | Records available UNTIL 15^th^ October 2011. Censored N=1,572  Or if mother or father deceased <10 yr |
| **Family Structure** |  |
| Mother age <20 years at child's birth | Complete |
| Father age <25 years or >=45 years at child's birth | Father not registered N=5,834  Father known, age missing N=325 |
| Child’s birth order 4^th^ or greater | Complete |
| Family size at child’s 10^th^ birthday:  One, three or four children | Complete |
| Two children | Complete |
| Five or more children | Complete |
| Mother not partnered at time of child’s birth | Complete |
| **Area Level Socio-economic/demographic** **Environment** |  |
| Value of index of socio-economic disadvantage in lowest quintile  Census-year-specific decile allocated for Census Collection District (CCD) of that  year according to mapping:  Census Birth years  1986 1980 – 1987  1991 1988 – 1993  1996 1994 – 1998  2001 1999 – 2001 | Unable to be classified N=825 |
| Remoteness classification: remote, very remote or unknown  Remoteness value in 2001 allocated by CCD identifier in 2001. ^[[3]](#footnote-3)^  Some aggregation of missing values done at postcode level. | Unable to be classified N=68 |
|  | Missing CCD2001 ***and*** no postal area aggregation N=353^[[4]](#footnote-4)^. Additional missing numbers due to statistics not provided at CCD level: |
| Percentage of persons of Aboriginal or Torres Strait Islander descent>=25% | N=202 |
| Percentage of persons Australian born <=75% | N=211 |
| Percentage of persons never married >=50% | N=211 |
| Percentage of one parent families >=20% | N=255 |
| Unemployment rate >=5% | N=202 |
| Percentage of persons living same residence as 5 years prior <40% | N=202 |
| Percentage of persons living same residence as 1 year prior <70% | N=202 |
| Percentage of persons living in semi-detached residences >=10% | N=213 |
| Percentage of persons living in residences that are flats >=5% | N=213 |
| Percentage of persons living in rented residences >=30% | N=216 |
| Percentage of persons who do not speak English well between 1 and 2% | N=202 |
| Has high ethnic heterogeneity | N=202 |
| Total crime - arrest rate per capita in 2002 >=50 per 1,000 residents  Values per Local Government Area (LGA) in 2002 allocated by 2001 LGA  Some missing 2001 LGA mapped by postcode (which is a sub-unit of LGA) | N=5 |
| Has more than minimal level of SEIFA inequality  Distribution of (2001 SEIFA value for CCD 2001 identifier) over postal areas^[[5]](#footnote-5)^ | N=8 |
| **Family Level Sociodemographic Status** |  |
| Father unknown / not registered at birth | Complete |
| Father in lower skill occupation or unemployed at time of child’s birth | Father not registered N=5,834  Father known, occupation missing N=6,186 |
| Mother born in low-income country or in Western Australia | Missing or irreconcilable data N=834 |
| Child of Aboriginal or Torres Strait Islander descent | Complete |

## Supplementary Table S2. Adversity exposures for children in development sample excluded or censored due to death. Counts and percentages.

|  | **Age child deceased** | | | | | |
| --- | --- | --- | --- | --- | --- | --- |
| **Adversity exposure** | **< 2 days**  **(excluded)** | | **>=2 days, <10 years**  **(excluded)** | | **>=10 years, before Sz diagnosis or before end of follow-up**  **(censored)** | |
|  | **N= 418** | **%** | **N= 840** | **%** | **N=**  **967** | **95%CI** |
| **Discontinuity in Parenting** |  |  |  |  |  |  |
| Mother hospitalised >= 8 days when child aged 15 days -<1 year | 45 | *10.8* | 34 | *4* | 20 | *2.1* |
| Mother hospitalised >= 8 days when child aged 1 -<5 years | 180 | *43.1* | 331 | *39.4* | 244 | *25.2* |
| Mother hospitalised >= 8 days when child aged 5 years -<10 years | 79 | *18.9* | 156 | *18.6* | 140 | *14.5* |
| Child hospitalised >= 8 days when child aged 15 days -<1 year |  |  | 149 | *17.7* | 85 | *8.8* |
| Child hospitalised >= 8 days when child aged 1 -<5years |  |  | 93 | *11.1* | 93 | *9.6* |
| Child hospitalised >= 8 days when child aged 5 years -<10 years |  |  | 37 | *4.4* | 79 | *8.2* |
| Father hospitalised >= 8 days when child aged 0 days -<1 year | <5 | *<1* | 8 | *1* | 15 | *1.6* |
| Father hospitalised >= 8 days when child aged 1 -<5 years | 14 | *3.3* | 30 | *3.6* | 47 | *4.9* |
| Father hospitalised >= 8 days when child aged 5 years -<10 years | 17 | *4.1* | 49 | *5.8* | 57 | *5.9* |
| Deceased parents, either or both, before child aged <10 years | <5 | *<1* | 27 | *3.2* | 23 | *2.4* |
| Any placement in foster care before child aged <10 years |  |  | <5 | *<1* | 6 | *0.6* |
| Any incarcerations for mother when child aged <10 years | 5 | *1.2* | 16 | *1.9* | 12 | *1.2* |
| Any incarcerations for father when child aged 0 days -<1 year | <5 | *<1* | 12 | *1.4* | 13 | *1.3* |
| Any incarcerations for father when child aged 1 -<5 years | 11 | *2.6* | 25 | *3* | 27 | *2.8* |
| Any incarcerations for father when child aged 5 years -<10 years | 10 | *2.4* | 24 | *2.9* | 42 | *4.3* |
| **Family Functioning** |  |  |  |  |  |  |
| Child protection contact before child aged 10 years:  Child or sibling victim of >=1 substantiated abuse | 15 | *3.6* | 62 | *7.4* | 48 | *5.0* |
| Child or sibling subject of >=1 unsubstantiated notification, no substantiated cases | 20 | *4.8* | 59 | *7.0* | 85 | *8.8* |
| Neither child nor siblings subject of any notifications | 383 | *91.6* | 719 | *85.6* | 834 | *86.2* |
| Any parental corrective services contact before child aged 10 years | 46 | *11.0* | 128 | *15.2* | 143 | *14.8* |
| **Family Structure** |  |  |  |  |  |  |
| Mother age <20 years at child's birth | 32 | *7.7* | 75 | *8.9* | 98 | *10.1* |
| Father age <25 years or >=45 years at child's birth | 107 | *25.6* | 237 | *28.2* | 273 | *28.2* |
| Child’s birth order 4^th^ or greater | 64 | *15.3* | 129 | *15.4* | 111 | *11.5* |
| Family size at child’s 10^th^ birthday:  One, three or four children | 260 | *62.2* | 526 | *62.6* | 524 | *54.2* |
| Two children | 56 | *13.4* | 123 | *14.6* | 331 | *34.2* |
| Five or more children | 102 | *24.4* | 191 | *22.7* | 112 | *11.6* |
| Mother not partnered at time of child’s birth | 368 | *88.0* | 715 | *85.1* | 834 | 86.2 |
| **Area Level Socio-economic/demographic** **Environment^a^** |  |  |  |  |  |  |
| Value of index of socio-economic disadvantage in lowest quintile | 102 | *24.4* | 227 | *27.0* | 222 | *23.0* |
| Remoteness classification: remote, very remote, or unknown | 62 | *14.8* | 149 | *17.7* | 178 | *18.4* |
| Percentage of persons of Aboriginal or Torres Strait Islander descent >=25% | 16 | *3.8* | 56 | *6.7* | 48 | *5.0* |
| Percentage of persons Australian born <=75% | 302 | *72.4* | 540 | *64.5* | 654 | *67.7* |
| Percentage of persons never married >=50% | 14 | *3.4* | 39 | *4.7* | 40 | *4.1* |
| Percentage of one parent families >=20% | 135 | *32.4* | 251 | *30.0* | 284 | *29.4* |
| Unemployment rate >=5% | 342 | *82.0* | 642 | *76.7* | 749 | *77.5* |
| Percentage of persons living same residence as 5 years prior <40% | 88 | *21.1* | 207 | *24.7* | 236 | *24.4* |
| Percentage of persons living same residence as 1 year prior <70% | 94 | *22.5* | 194 | *23.2* | 220 | *22.8* |
| Percentage of persons living in semi-detached residences >=10% | 107 | *25.7* | 188 | *22.5* | 238 | *24.6* |
| Percentage of persons living in residences that are flats >=5% | 87 | *20.9* | 147 | *17.6* | 167 | *17.3* |
| Percentage of persons living in rented residences >=30% | 131 | *31.4* | 294 | *35.1* | 344 | *35.6* |
| Percentage of persons who do not speak English well between 1 and 2% | 28 | *6.7* | 38 | *4.5* | 45 | *4.7* |
| Total crime - arrest rate in 2002 >=50 per 1,000 residents | 50 | *12.0* | 105 | *12.5* | 132 | *13.7* |
| Has high ethnic heterogeneity | 409 | *98.1* | 810 | *96.8* | 937 | *97.0* |
| Has more than minimal level of SEIFA inequality | 38 | *9.1* | 127 | *15.2* | 138 | *14.3* |
| **Family Level Sociodemographic Status** |  |  |  |  |  |  |
| Father unknown / not registered at birth | 39 | *9.3* | 87 | *10.4* | 67 | *6.9* |
| Father in lower skill occupation or unemployed at time of child’s birth | 76 | *20.1* | 163 | *21.6* | 186 | *20.7* |
| Mother born in low-income country or in Western Australia | 239 | *57.2* | 530 | *63.1* | 634 | *65.6* |
| Child of Aboriginal or Torres Strait Islander descent | 58 | *13.9* | 135 | *16.1* | 153 | *15.8* |

## Supplementary Table S3. Exposure to adversity in validation samples. Number and percentage.

|  | **Validation sample 1** | | | | **Validation sample 2** | | | |
| --- | --- | --- | --- | --- | --- | --- | --- | --- |
| **Adversity exposure** | **Child with diagnosis of Sz** | | **Total** | | **Child with diagnosis of Sz** | | **Total** | |
|  | **N=472** | **%** | **N=128,691** | **%** | **N=492** | **%** | **N=128,691** | **%** |
| **Discontinuity in Parenting** |  |  |  |  |  |  |  |  |
| Mother hospitalised >= 8 days when child aged 15 days -<1 year | 14 | 3 | 1188 | 0.9 | 10 | 2 | 1206 | 0.9 |
| Mother hospitalised >= 8 days when child aged 1 -<5 years | 138 | 29.2 | 22993 | 17.9 | 142 | 28.9 | 22992 | 17.9 |
| Mother hospitalised >= 8 days when child aged 5 years -<10 years | 93 | 19.7 | 12380 | 9.6 | 109 | 22.2 | 12417 | 9.6 |
| Child hospitalised >= 8 days when child aged 15 days -<1 year | 32 | 6.8 | 3098 | 2.4 | 26 | 5.3 | 3061 | 2.4 |
| Child hospitalised >= 8 days when child aged 1 -<5years | 36 | 7.6 | 3230 | 2.5 | 37 | 7.5 | 3175 | 2.5 |
| Child hospitalised >= 8 days when child aged 5 years -<10 years | 13 | 2.8 | 1496 | 1.2 | 13 | 2.6 | 1580 | 1.2 |
| Father hospitalised >= 8 days when child aged 0 days -<1 year | 5 | 1.1 | 727 | 0.6 | 5 | 1 | 706 | 0.5 |
| Father hospitalised >= 8 days when child aged 1 -<5 years | 20 | 4.2 | 3695 | 2.9 | 20 | 4.1 | 3637 | 2.8 |
| Father hospitalised >= 8 days when child aged 5 years -<10 years | 31 | 6.6 | 4515 | 3.5 | 29 | 5.9 | 4606 | 3.6 |
| Deceased parents, either or both, before child aged <10 years | 8 | 1.7 | 1545 | 1.2 | 11 | 2.2 | 1475 | 1.1 |
| Any placement in foster care before child aged <10 years | 12 | 2.5 | 506 | 0.4 | 8 | 1.6 | 519 | 0.4 |
| Any incarcerations for mother when child aged <10 years | 10 | 2.1 | 478 | 0.4 | 9 | 1.8 | 479 | 0.4 |
| Any incarcerations for father when child aged 0 days -<1 year | 12 | 2.5 | 727 | 0.6 | 10 | 2 | 708 | 0.6 |
| Any incarcerations for father when child aged 1 -<5 years | 21 | 4.4 | 1735 | 1.3 | 20 | 4.1 | 1699 | 1.3 |
| Any incarcerations for father when child aged 5 years -<10 years | 10 | 2.1 | 1805 | 1.4 | 18 | 3.7 | 1697 | 1.3 |
| **Family Functioning** |  |  |  |  |  |  |  |  |
| Child protection contact before child aged 10 years:  Child or sibling victim of >=1 substantiated abuse | 34 | 7.2 | 2556 | 2 | 33 | 6.7 | 2675 | 2.1 |
| Child or sibling subject of >=1 unsubst. notification, no substantiated cases | 47 | 10 | 6074 | 4.7 | 52 | 10.6 | 5916 | 4.6 |
| Any parental corrective services contact before child aged 10 years | 81 | 17.2 | 10813 | 8.4 | 77 | 15.7 | 10780 | 8.4 |
| **Family Structure** |  |  |  |  |  |  |  |  |
| Mother age <20 years at child's birth | 44 | 9.3 | 6348 | 4.9 | 49 | 10 | 6287 | 4.9 |
| Father age <25 years or >=45 years at child's birth | 136 | 28.8 | 22922 | 17.8 | 142 | 28.9 | 22670 | 17.6 |
| Child’s birth order 4^th^ or greater | 69 | 14.6 | 11599 | 9 | 57 | 11.6 | 11730 | 9.1 |
| Family size at child’s 10^th^ birthday:  One, three or four children | 256 | 54.3 | 69757 | 54.2 | 263 | 53.5 | 69676 | 54.1 |
| Two children | 138 | 29.2 | 49313 | 38.3 | 162 | 32.9 | 49526 | 38.5 |
| Five or more children | 78 | 16.5 | 9621 | 7.5 | 67 | 13.6 | 9489 | 7.4 |
| Mother not partnered at time of child’s birth | 79 | 16.7 | 10303 | 8 | 78 | 15.9 | 10267 | 8 |
| **Area Level Socio-economic/demographic Environmenta** |  |  |  |  |  |  |  |  |
| Value of index of socio-economic disadvantage in lowest quintile | 130 | 27.5 | 24248 | 18.8 | 116 | 23.6 | 24233 | 18.8 |
| Remoteness classification: remote, very remote, or unknown | 66 | 14 | 14412 | 11.2 | 55 | 11.2 | 14274 | 11.1 |
| Percentage of persons of Aboriginal or Torres Strait Islander descent >=25% | 26 | 5.5 | 2931 | 2.3 | 17 | 3.5 | 2871 | 2.2 |
| Percentage of persons Australian born <=75% | 326 | 69.1 | 93236 | 72.4 | 361 | 73.4 | 93058 | 72.3 |
| Percentage of persons never married >=50% | 34 | 7.2 | 4378 | 3.4 | 25 | 5.1 | 4248 | 3.3 |
| Percentage of one parent families >=20% | 174 | 36.9 | 34353 | 26.7 | 151 | 30.7 | 34340 | 26.7 |
| Unemployment rate >=5% | 385 | 81.6 | 100283 | 77.9 | 399 | 81.1 | 100060 | 77.8 |
| Percentage of persons living same residence as 5 years prior <40% | 125 | 26.5 | 28925 | 22.5 | 112 | 22.8 | 28994 | 22.5 |
| Percentage of persons living same residence as 1 year prior <70% | 117 | 24.8 | 25394 | 19.7 | 114 | 23.2 | 25332 | 19.7 |
| Percentage of persons living in semi-detached residences >=10% | 148 | 31.4 | 30351 | 23.6 | 142 | 28.9 | 30247 | 23.5 |
| Percentage of persons living in residences that are flats >=5% | 109 | 23.1 | 22355 | 17.4 | 112 | 22.8 | 22661 | 17.6 |
| Percentage of persons living in rented residences >=30% | 183 | 38.8 | 37296 | 29 | 164 | 33.3 | 37442 | 29.1 |
| Percentage of persons who do not speak English well between 1 and 2% | 19 | 4 | 7498 | 5.8 | 70 | 14.2 | 16712 | 13 |
| Total crime - arrest rate per capita in 2002 >=50 | 69 | 14.6 | 16693 | 13 | 481 | 97.8 | 125416 | 97.5 |
| Has high ethnic heterogeneity | 457 | 96.8 | 125375 | 97.4 | 59 | 12 | 10301 | 8 |
| Has more than minimal level of SEIFA inequality | 61 | 12.9 | 10516 | 8.2 | 462 | 93.9 | 116006 | 90.1 |
| **Family Level Sociodemographic Status** |  |  |  |  |  |  |  |  |
| Father unknown / not registered at birth | 50 | 10.6 | 4301 | 3.3 | 46 | 9.3 | 4337 | 3.4 |
| Father in lower skill occupation or unemployed at time of child’s birth | 105 | 22.2 | 20079 | 15.6 | 83 | 16.9 | 19771 | 15.4 |
| Mother born in low-income country or in Western Australia | 315 | 66.7 | 76090 | 59.1 | 320 | 65 | 76052 | 59.1 |
| Child of Aboriginal or Torres Strait Islander descent | 92 | 19.5 | 9188 | 7.1 | 89 | 18.1 | 8916 | 6.9 |

## Supplementary Table S4. Development sample observations excluded from final model parameter estimation due to large influence. N=27 observations, all with schizophrenia diagnosis. Adversity exposure comparison with non-excluded observations.

|  | **Excluded observations** | | | **Non excluded observations** | | | |
| --- | --- | --- | --- | --- | --- | --- | --- |
| **Adversity Exposure** | | **Child with Sz, N=27** | | **Child  with Sz, N=629** | | **Child  NO Sz, N=170,932** | |
|  | | **N** | **%** | **N** | **%** | **N** | **%** |
| **Discontinuity in parenting** | |  |  |  |  |  |  |
| Mother hospitalised >= 8 days when child aged 15 days -<1 year | | <5 | <15 | 12 | 1.9 | 1630 | 1 |
| Mother hospitalised >= 8 days when child aged 1 -<5 years | | 17 | 63 | 205 | 32.6 | 30937 | 18.1 |
| Mother hospitalised >= 8 days when child aged 5 years -<10 years | | 14 | 51.9 | 110 | 17.5 | 16431 | 9.6 |
| Child hospitalised >= 8 days when child aged 15 days -<1 year | | 7 | 25.9 | 35 | 5.6 | 4124 | 2.4 |
| Child hospitalised >= 8 days when child aged 1 -<5years | | 5 | 18.5 | 39 | 6.2 | 4175 | 2.4 |
| Child hospitalised >= 8 days when child aged 5 years -<10 years | | <5 | <15 | 16 | 2.5 | 1964 | 1.1 |
| Father hospitalised >= 8 days when child aged 0 days -<1 year | | <5 | <15 | 9 | 1.4 | 945 | 0.6 |
| Father hospitalised >= 8 days when child aged 1 -<5 years | | 8 | 29.6 | 30 | 4.8 | 4818 | 2.8 |
| Father hospitalised >= 8 days when child aged 5 years -<10 years | | <5 | <15 | 38 | 6 | 6004 | 3.5 |
| Deceased parents, either or both, before child aged <10 years | | <5 | <5 | 11 | 1.7 | 2065 | 1.2 |
| Any placement in foster care before child aged <10 years | | 5 | 18.5 | 11 | 1.7 | 692 | 0.4 |
| Any incarcerations for mother when child aged <10 years | | <5 | <15 | 8 | 1.3 | 646 | 0.4 |
| Any incarcerations for father when child aged 0 days -<1 year | | <5 | <15 | 10 | 1.6 | 882 | 0.5 |
| Any incarcerations for father when child aged 1 -<5 years | | 6 | 22.2 | 19 | 3 | 2214 | 1.3 |
| Any incarcerations for father when child aged 5 years -<10 years | | 6 | 22.2 | 19 | 3 | 2308 | 1.4 |
| **Family functioning** | |  |  |  |  |  |  |
| Child protection contact before child aged 10 years:  Child or sibling victim of >=1 substantiated abuse | | 7 | 25.9 | 38 | 6 | 3388 | 2 |
| Child or sibling subject of >=1 unsubstantiated   notification, no substantiated cases | | 6 | 22.2 | 55 | 8.7 | 8046 | 4.7 |
| Neither child nor siblings subject of any notifications | | *14* | *51.9* | 536 | 85.3 | 159498 | 93.3 |
| Any parental corrective services contact before child aged 10 years | | *12* | *48* | 78 | 12.4 | 14291 | 8.4 |
| **Family structure** | |  |  |  |  |  |  |
| Mother age <20 years at child's birth | | 14 | 51.9 | 53 | 8.4 | 8418 | 4.9 |
| Father age <25 years or >=45 years at child's birth | | 8 | 29.6 | 183 | 29.1 | 30284 | 17.7 |
| Child’s birth order 4^th^ or greater | | 3 | 11.1 | 78 | 12.4 | 15442 | 9 |
| Family size at child’s 10^th^ birthday:  One, three or four children | | *19* | *70.4* | 364 | 57.9 | 92540 | 54.1 |
| Two children | | <5 | <15 | 180 | 28.6 | 65776 | 38.5 |
| Five or more children | | 5 | 18.5 | 85 | 13.5 | 12616 | 7.4 |
| Mother not partnered at time of child’s birth | | 9 | 33.3 | 96 | 15.3 | 13137 | 7.7 |
| **Area Level Socio-economic/demographic** **Environment** | |  |  |  |  |  |  |
| Value of index of socio-economic disadvantage in lowest quintile | | 14 | 51.9 | 168 | 26.6 | 32494 | 19 |
| Remoteness classification: remote, very remote, or unknown | | 9 | 33.3 | 90 | 14.3 | 19159 | 11.2 |
| Percentage of persons of Aboriginal or Torres Strait Islander descent >=25% | | <5 | <15 | 27 | 4.3 | 3745 | 2.2 |
| Percentage of persons Australian born <=75% | | 14 | 51.9 | 467 | 74 | 124098 | 72.6 |
| Percentage of persons never married >=50% | | <5 | <15 | 44 | 7 | 5880 | 3.4 |
| Percentage of one parent families >=20% | | 14 | 51.9 | 204 | 32.3 | 45679 | 26.7 |
| Unemployment rate >=5% | | 17 | 63 | 512 | 81.1 | 133588 | 78.2 |
| Percentage of persons living same residence as 5 years prior <40% | | 10 | 37 | 158 | 25 | 38710 | 22.6 |
| Percentage of persons living same residence as 1 year prior <70% | | 10 | 37 | 159 | 25.2 | 34021 | 19.9 |
| Percentage of persons living in semi-detached residences >=10% | | 12 | 44.4 | 182 | 28.8 | 40282 | 23.6 |
| Percentage of persons living in residences that are flats >=5% | | 6 | 22.2 | 146 | 23.1 | 30214 | 17.7 |
| Percentage of persons living in rented residences >=30% | | 17 | 63 | 236 | 37.4 | 50010 | 29.3 |
| Percentage of persons who do not speak English well between 1 and 2% | | <5 | <15 | 103 | 16.3 | 21896 | 12.8 |
| Total crime - arrest rate per capita in 2002 >=50 | | <5 | <15 | 621 | 98.4 | 167004 | 97.7 |
| Has high ethnic heterogeneity | | 7 | 25.9 | 85 | 13.5 | 13696 | 8 |
| Has more than minimal level of SEIFA inequality | | 20 | 74.1 | 579 | 91.8 | 154697 | 90.5 |
| **Family Level Sociodemographic Status** | |  |  |  |  |  |  |
| Father unknown / not registered at birth | | <5 | <15 | 49 | 7.8 | 5833 | 3.4 |
| Father in lower skill occupation or unemployed at time of child’s birth | | 11 | 40.7 | 129 | 20.4 | 26701 | 15.6 |
| Mother born in low-income country or in Western Australia | | 21 | 77.8 | 413 | 65.5 | 101412 | 59.3 |
| Child of Aboriginal or Torres Strait Islander descent | | 14 | 51.9 | 117 | 18.5 | 12156 | 7.1 |

## Supplementary Table S5. Parameter estimates derived from development sample including observations with large influence. Comparison with optimum models. Hazard ratios.

|  | **Influential observations included** | **Influential observations excluded** |
| --- | --- | --- |
| **Model M1 - Exposure to *Discontinuity in parenting* and risk of schizophrenia. N= 2 excluded** | **HR** | **HR** |
| Mother hospitalised >= 8 days when child aged 1 - <5 years | 1.55 | 1.55 |
| Mother hospitalised >= 8 days when child aged 5 years -<10 years | 1.40 | 1.39 |
| Child hospitalised >= 8 days when child aged 15 days -<1 year | 1.72 | 1.67 |
| Child hospitalised >= 8 days when child aged 1 -<5 years | 1.38 | 1.40 |
| Child hospitalised >= 8 days when child aged 5 years -<10 years | 1.38 | 1.39 |
| Father hospitalised >= 8 days when child aged 1 -<5 years | 1.46 | 1.43 |
| Father hospitalised >= 8 days when child aged 5 years -<10 years | 1.36 | 1.35 |
| Any placement in foster care before child aged <10 years | 4.95 | 4.71 |
| Any incarcerations for father when child aged 1 - <5 years | 1.72 | 1.80 |
| Any incarcerations for father when child aged 5 years - <10 years | 1.65 | 1.55 |
| **Model M2 - Exposure to *Family functioning* and risk of schizophrenia. N=0 excluded.** |  |  |
| *no observations were excluded from optimum domain model* |  |  |
| **Model M3 - Exposure to *Family structure* and risk of schizophrenia. N=12 excluded.** |  |  |
| Father age < 25 years or >=45 years at child's birth | 1.51 | 1.55 |
| Family size at child’s 10^th^ birthday:  One, three or four children | 1.35 | 1.32 |
| Two children | 2.36 | 2.34 |
| Five or more children | 1.72 | 1.67 |
| Mother not partnered at time of child’s birth | 1.51 | 1.55 |
| **Model M4 - Exposure to *Area level socio-economic/demographic environment* and risk of schizophrenia. N=7 excluded** |  |  |
| Value of index of socio-economic disadvantage in lowest quintile | 1.38 | 1.39 |
| Percentage of persons never married >=50% | 1.57 | 1.54 |
| Percentage of one parent families >=20% | 1.13 | 1.14 |
| Percentage of persons living in residences that are flats >=5% | 1.20 | 1.21 |
| Percentage of persons who do not speak English well between 1 and 2% | 1.30 | 1.31 |
| Has high ethnic heterogeneity | 1.40 | 1.40 |
| Missing CCD information and unable to interpolate domain exposures | 2.53 | 1.00 |
| **Model M5 - Exposure to *Family level sociodemographic status* and risk of schizophrenia. N=0 excluded** |  |  |
| *no observations were excluded from optimum domain model* |  |  |
| **Global model. N=27 excluded** |  |  |
| Discontinuity in parenting calculated as log (HR) of M1 | 2.03 | 1.93 |
| Family functioning calculated as log (HR) of M2 | 1.92 | 1.90 |
| Family structure calculated as log (HR) of M3 | 1.58 | 1.67 |
| Area level socio-economic/demographic environment calculated as log (HR) of M4 | 1.95 | 1.99 |
| Family level sociodemographic status calculated as log (HR) of M5 | 1.52 | 1.52 |
| Interaction: Discontinuity in parenting and Area level socio-economic/demographic environment  calculated as {log (HR) of M1} x {log (HR) of M4} | 0.68 | 0.58 |
| Interaction: Family functioning and Family structure  calculated as {log (HR) of M2} x {log (HR) of M3} | 0.67 | 0.64 |

## Supplementary Table S6. Elementary validations for models. Robustness of estimated Cox model hazard ratios - comparison with estimated logistic regression odds ratios (Ratios and 95% Confidence intervals). Proportional hazards validity – test for non-zero correlation of Schoenfeld residuals with time (P values).

|  | **Cox Proportional Hazards** | **Logistic Regression** | **Proportional hazards** |
| --- | --- | --- | --- |
| **Model M1 - Exposure to *Discontinuity in parenting* and risk of schizophrenia** | **HR 95%CI** | **OR 95%CI** | **P value** |
| Mother hospitalised >= 8 days when child aged 1 - <5 years | 1.56 (1.32-1.84) | 1.81 (1.52-2.14) | 0.976 |
| Mother hospitalised >= 8 days when child aged 5 years -<10 years | 1.38 (1.13-1.70) | 1.49 (1.21-1.84) | 0.821 |
| Child hospitalised >= 8 days when child aged 15 days -<1 year | 1.67 (1.18-2.37) | 1.66 (1.17-2.36) | 0.083 |
| Child hospitalised >= 8 days when child aged 1 -<5 years | 1.41 (1.00-1.99) | 1.53 (1.08-2.17) | 0.638 |
| Child hospitalised >= 8 days when child aged 5 years -<10 years | 1.39 (0.86-2.23) | 1.46 (0.90-2.36) | 0.907 |
| Father hospitalised >= 8 days when child aged 1 -<5 years | 1.43 (1.02-2.01) | 1.53 (1.08-2.16) | 0.107 |
| Father hospitalised >= 8 days when child aged 5 years -<10 years | 1.34 (0.97-1.87) | 1.37 (0.99-1.91) | 0.121 |
| Any placement in foster care before child aged <10 years | 4.70 (2.75-8.01) | 3.27 (1.90-5.62) | 0.448 |
| Any incarcerations for father when child aged 1 - <5 years | 1.80 (1.08-2.99) | 1.71 (1.02-2.86) | 0.928 |
| Any incarcerations for father when child aged 5 years - <10 years | 1.56 (0.93-2.62) | 1.42 (0.84-2.40) | 0.893 |
| **Model M2 - Exposure to *Family functioning* and risk of schizophrenia** |  |  |  |
| Child protection contact before child aged 10 years:  Child or sibling subject of >=1 notification, no substantiated abuse | 2.17 (1.64-2.86) | 2.09 (1.58-2.75) | 0.094 |
| Child or sibling victim of >=1 substantiated abuse | 3.51 (2.55-4.82) | 3.56 (2.58-4.91) | 0.075 |
| No notification for any child or sibling within family | Reference | Reference | - |
| Any parental corrective services contact before child aged 10 years | 1.80 (1.42-2.29) | 1.47 (1.16-1.87) | 0.728 |
| **Model M3 - Exposure to *Family structure* and risk of schizophrenia** |  |  |  |
| Father age < 25 years or >=45 years at child's birth | 1.56 (1.29-1.88) | 1.61 (1.34-1.95) | 0.678 |
| Family size at child’s 10^th^ birthday:  One, three or four children | 1.32 (1.11-1.58) | 1.38 (1.15-1.64) | 0.189 |
| Two children | Reference | Reference | - |
| Five or more children | 2.35 (1.82-3.02) | 2.40 (1.86-3.10) | 0.288 |
| Mother not partnered at time of child’s birth | 1.66 (1.31-2.10) | 1.63 (1.29-2.06) | 0.087 |
| **Model M4 - Exposure to *Area level socio-economic/demographic environment* and risk of schizophrenia** |  |  |  |
| Value of index of socio-economic disadvantage in lowest quintile | 1.39 (1.13-1.70) | 1.31 (1.07-1.61) | 0.951 |
| Percentage of persons never married >=50% | 1.53 (1.12-2.11) | 1.58 (1.15-2.17) | 0.377 |
| Percentage of one parent families >=20% | 1.13 (0.95-1.35) | 1.13 (0.94-1.35) | 0.348 |
| Percentage of persons living in residences that are flats >=5% | 1.20 (1.00-1.46) | 1.26 (1.04-1.52) | 0.377 |
| Percentage of persons who do not speak English well between 1 and 2% | 1.31 (1.06-1.61) | 1.33 (1.08-1.64) | 0.245 |
| Has high ethnic heterogeneity | 1.40 (1.09-1.79) | 1.48 (1.15-1.89) | 0.397 |
| **Model M5 - Exposure to *Family level sociodemographic status* and risk of schizophrenia** |  |  |  |
| Father unknown / not registered at birth | 1.63 (1.20-2.23) | 1.66 (1.22-2.27) | 0.158 |
| Father in lower skill occupation or unemployed at time of child’s birth | 1.36 (1.12-1.66) | 1.32 (1.09-1.61) | 0.602 |
| Mother born in low-income country or in Western Australia | 1.18 (1.00-1.39) | 1.17 (1.00-1.39) | 0.326 |
| Child of Aboriginal or Torres Strait Islander descent | 2.81 (2.27-3.47) | 2.75 (2.23-3.41) | 0.898 |
| **Global model** |  |  |  |
| Discontinuity in parenting calculated as log (HR) of M1 | 1.93 (1.52-2.45) | 2.27 (1.80-2.86) | 0.827 |
| Family functioning calculated as log (HR) of M2 | 1.90 (1.36-2.66) | 1.55 (1.10-2.17) | 0.913 |
| Family structure calculated as log (HR) of M3 | 1.66 (1.31-2.11) | 1.78 (1.40-2.25) | 0.692 |
| Area level socio-economic/demographic environment calculated as log (HR) of M4 | 1.99 (1.46-2.70) | 2.05 (1.51-2.78) | 0.480 |
| Family level sociodemographic status calculated as log (HR) of M5 | 1.53 (1.26-1.86) | 1.47 (1.21-1.79) | 0.962 |
| Interaction: Discontinuity in parenting and Area level socio-economic/demographic environment  calculated as {log (HR) of M1} x {log (HR) of M4} | 0.58 (0.37-0.90) | 0.55 (0.36-0.85) | 0.546 |
| Interaction: Family functioning and Family structure  calculated as {log (HR) of M2} x {log (HR) of M3} | 0.65 (0.45-0.93) | 0.64 (0.44-0.93) | 0.551 |

## Supplementary Table S7. Risk of developing schizophrenia following exposure to adversity across five domains. Alternative Global models. Hazard ratios and 95%Confidence intervals.

| **Alternative global model G_1** | **HR** | **95%CI** |
| --- | --- | --- |
| A1 Discontinuity in parenting -calculated as log (HR) of M1 | 1.58 | (1.32-1.89) |
| A2 Family functioning -calculated as log (HR) of M2 | 1.34 | (1.09-1.65) |
| A3 Family structure -calculated as log (HR) of M3 | 1.54 | (1.24-1.91) |
| A4 Area level socio-economic/demographic environment -calculated as log (HR) of M4 | 1.60 | (1.23-2.07) |
| A5 Family level sociodemographic status   -calculated as log (HR) of M5 | 1.49 | (1.22-1.81) |
| **Alternative global model G_2** | **HR** | **95%CI** |
| A1 Discontinuity in parenting -calculated as log (HR) of M1_i^a^ | 2.29 | (1.71-3.08) |
| A2 Family functioning -calculated as log (HR) of M2 | 2.37 | (1.62-3.48) |
| A3 Family structure -calculated as log (HR) of M3 | 1.65 | (1.29-2.1) |
| A4 Area level socio-economic/demographic environment -calculated as log (HR) of M4_i^a^ | 1.78 | (1.39-2.28) |
| A5 Family level sociodemographic status   -calculated as log (HR) of M5 | 1.66 | (1.28-2.17) |
| A1.A2 Interaction – {log (HR) of M1_i} x {log (HR) of M2} | 0.69 | (0.47-1.004) |
| A1.A5 Interaction – {log (HR) of M1_i} x {log (HR) of M5} | 0.71 | (0.5-1.01) |
| A2.A3 Interaction – {log (HR) of M2} x {log (HR) of M3} | 0.56 | (0.36-0.87) |

## ^a^Alternative model parameterisations for risk of developing schizophrenia following exposure to adversity across domains A1 – Discontinuity in parenting and A4 – Area level socio-economic/demographic environment. Hazard ratios and 95% Confidence intervals.

| **Model M1_i - Exposure to *Discontinuity in parenting* and risk of schizophrenia** | **HR** | **95%CI** |
| --- | --- | --- |
| Mother hospitalised >= 8 days when child aged 1 - <5 years | 1.67 | 1.39 – 2.02 |
| Mother hospitalised >= 8 days when child aged 5 years -<10 years | 1.72 | 1.31 – 2.25 |
| Child hospitalised >= 8 days when child aged 15 days -<1 year | 1.89 | 1.25 – 2.86 |
| Child hospitalised >= 8 days when child aged 1 -<5 years | 1.65 | 1.12 – 2.43 |
| Child hospitalised >= 8 days when child aged 5 years -<10 years | 1.35 | 0.82 – 2.22 |
| Father hospitalised >= 8 days when child aged 1 -<5 years | 1.30 | 0.90 – 1.87 |
| Father hospitalised >= 8 days when child aged 5 years -<10 years | 1.35 | 0.96 – 1.89 |
| Any placement in foster care before child aged <10 years | 3.11 | 1.58 – 6.11 |
| Any incarcerations for father when child aged 1 - <5 years | 1.43 | 0.80 – 2.54 |
| Any incarcerations for father when child aged 5 years - <10 years | 1.57 | 0.90 – 2.76 |
| Interaction: Mother hospitalised >= 8 days when child aged 1 -<5 years   AND   Mother hospitalised >= 8 days when child aged 5 -<10 years | 0.64 | 0.43 – 0.97 |
| Interaction: Child hospitalised >= 8 days when child aged 15 days -<1 year  AND  Child hospitalised >= 8 days when child aged 5 years -<10 years | 0.58 | 0.26 – 1.29 |
| **Model M4_i - Exposure to *Area level socio-economic/demographic environment* and risk of schizophrenia** | **HR** | **95%CI** |
| Value of index of socio-economic disadvantage in lowest quintile | 1.57 | 1.28 – 1.94 |
| Percentage of persons never married >=50% | 2.24 | 1.44 – 3.48 |
| Percentage of persons living in residences that are flats >=5% | 1.28 | 1.05 – 1.56 |
| Percentage of persons who do not speak English well between 1 and 2% | 1.45 | 1.15 – 1.83 |
| Has high ethnic heterogeneity | 1.32 | 1.02 – 1.70 |
| Interaction: Percentage of persons never married >=50%   AND   Percentage of persons living in residences that are flats >=5% | 0.46 | 0.24 – 0.87 |
| Interaction: Value of index of socio-economic disadvantage in lowest quintile   AND   Percentage of persons who do not speak English well between 1 and 2% | 0.58 | 0.34 – 1.01 |

## Supplementary Table S8. Alternative Global Cox model parameterisations describing risk of schizophrenia following exposure to adversity. Harrell’s Concordance statistics.

|  | **Harrell’s Concordance** | |
| --- | --- | --- |
| **Model describing risk of schizophrenia following exposure to adversity** | **Validation set 1** | **Validation set 2** |
| Global model G_1, as defined in Table S8 | 0.655 | 0.624 |
| Global model G_2, as defined in Table S8 | 0.658 | 0.623 |

## Supplementary Figure S1. Distributions of predicted log hazard ratios for five domain models and Global model, derived from development sample.
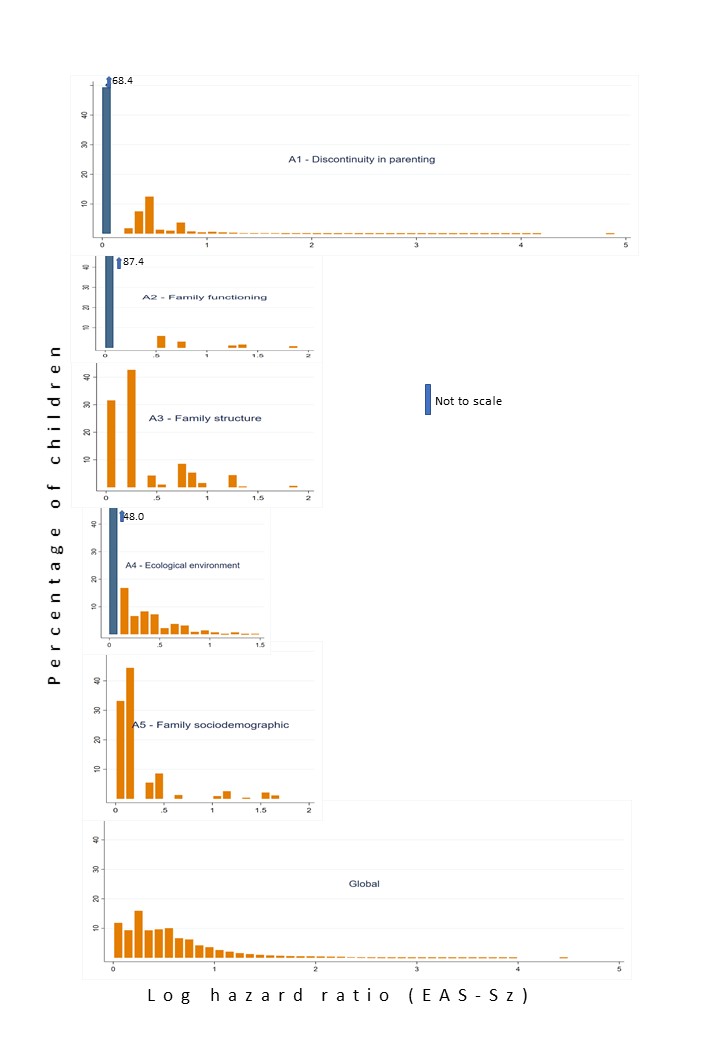


1. Children born before 1^st^ April 1981 have censored observations for very early life. Any foster placements after this date, up to a child’s 10^th^ birthday, will still contribute to a recorded exposure for that child. [↑](#footnote-ref-1)
2. No cases have completely missing data. Eg, for a child born in 1988, the only missing data is for their 1^st^ year of life. Any substantiations after that, up to their 10^th^ birthday, still contribute to a recorded exposure for that child. Additionally, maternal sibling substantiations contribute to the index child’s exposure. It is estimated we are missing ~19.8% of total expected flags, based on numbers observed for children born 1989 onwards. [↑](#footnote-ref-2)
3. Remoteness classifications only available from 2001 onwards, hence allocation to birth record based on 2001 CCD identifier of residence at birth. [↑](#footnote-ref-3)
4. Postcode information allowed imputation where smaller level CCD identifiers not available for N=23,839 [↑](#footnote-ref-4)
5. Different mechanism to calculate than that used to allocate SEIFA quintile. The inequality approach is necessarily more blunt (all based on 2001 values). [↑](#footnote-ref-5)
